# Amyloid Beta Precursor Protein and Prion Protein Have a Conserved Interaction Affecting Cell Adhesion and CNS Development

**DOI:** 10.1371/journal.pone.0051305

**Published:** 2012-12-07

**Authors:** Darcy M. Kaiser, Moulinath Acharya, Patricia L. A. Leighton, Hao Wang, Nathalie Daude, Serene Wohlgemuth, Beipei Shi, W. Ted Allison

**Affiliations:** 1 Centre for Prions and Protein Folding Diseases, University of Alberta, Edmonton, Alberta, Canada; 2 Department of Biological Sciences, University of Alberta, Edmonton, Alberta, Canada; 3 Department of Medical Genetics, University of Alberta, Edmonton, Alberta, Canada; Massachusetts General Hospital, United States of America

## Abstract

Genetic and biochemical mechanisms linking onset or progression of Alzheimer Disease and prion diseases have been lacking and/or controversial, and their etiologies are often considered independent. Here we document a novel, conserved and specific genetic interaction between the proteins that underlie these diseases, amyloid-β precursor protein and prion protein, APP and PRP, respectively. Knockdown of *APP* and/or *PRNP* homologs in the zebrafish (*appa*, *appb*, *prp1*, and *prp2*) produces a dose-dependent phenotype characterized by systemic morphological defects, reduced cell adhesion and CNS cell death. This genetic interaction is surprisingly exclusive in that *prp1* genetically interacts with zebrafish *appa*, but not with *appb,* and the zebrafish paralog *prp2* fails to interact with *appa*. Intriguingly, *appa* & *appb* are largely redundant in early zebrafish development yet their abilities to rescue CNS cell death are differentially contingent on *prp1* abundance. Delivery of human *APP* or mouse *Prnp* mRNAs rescue the phenotypes observed in *app*-*prp*-depleted zebrafish, highlighting the conserved nature of this interaction. Immunoprecipitation revealed that human APP and PrP^C^ proteins can have a physical interaction. Our study reports a unique *in vivo* interdependence between APP and PRP loss-of-function, detailing a biochemical interaction that considerably expands the hypothesized roles of PRP in Alzheimer Disease.

## Introduction

Amyloid-β precursor protein (APP) is a highly conserved type Ι transmembrane protein that liberates Aβ peptides into the extracellular space when it is sequentially cleaved by β- and γ-secretases [1]. These Aβ peptides can aggregate into soluble oligomers or fibrillar assembles with the tinctoral properties of amyloids. This is thought to be the initiating pathological event in Alzheimer Disease [1], [2]. Cellular prion protein (PrP^C^) is a conserved GPI-anchored membrane protein that, when misfolded into an aberrant conformation (PrP^Sc^), is able to recruit and template the misfolding of normal PrP^C^. This initiates the pathological events in Creutzfeldt-Jakob disease, scrapie, and bovine spongiform encephalopathy [3].

Intense interest has recently focused on a high-affinity interaction between PrP^C^ and oligomerized Aβ [4]–[6] as a potential route to explain excitotoxicity or learning deficits during disease, though the physiological effects of this interaction on synaptogenesis and long-term potentiation have been controversial. Considering the possible biochemical interaction between App holoprotein and PrP^C^ suggested by interactome studies [7]–[9], and striking parallels in their cellular locations [10]–[12], overlapping expression [13], [14] and functions (cell adhesion, regeneration, metal homeostasis, neuroprotection, regulation of neuronal excitability [15]–[23]), we inferred that *APP* and *PRNP* may be in a common genetic pathway. We examined the functional significance and conservation of this hypothetical interaction via concerted knockdown of these proteins in zebrafish.

Zebrafish, *Danio rerio*, has two paralogs of *APP* and two paralogs of *PRNP*. Zebrafish paralogs both show ∼70% predicted identity to human APP, increasing to ∼90–100% identity in the regions that encode the amyloidogenic, transmembrane and intracellular domains [24], [25]. Zebrafish *appa* and *appb* are homologs of mammalian *APP*, and are less similar to mammalian APP-like proteins (*APLP1* & *APLP2*, of which zebrafish have further additional homologs not considered herein). The two zebrafish paralogs of *PRNP* are not similar to mammalian homologs at the level of amino acid identity, but share a substantial conservation of protein domain architecture [14]. Regardless, the ability of mammalian *APP* or *PRNP* to replace zebrafish homologues argues strongly for an impressive conservation of function (Results herein, see also [24], [26]).

Here we report an *in vivo* genetic interdependence of *APP* and *PRNP* homologues in zebrafish. The results delineate surprisingly specific interactions between gene paralogs, as *appa* shows a genetic interaction with *prp1*, whereas *appb* does not. Similarly, *appa* interacts with *prp1*, but not with *prp2*. Cell death and disrupted cell adhesion are evident when *appa* and *prp1* are disrupted. *appa* and *appb* are able to replace each other, and thus are largely redundant during zebrafish development; however these paralogs have divergent abilities to rescue CNS cell death based on the state of PrP abundance. Finally, our results demonstrate that mouse *Prnp* and human *APP* are both able to replace their zebrafish orthologs in the neurodevelopmental processes that require the observed APP-PrP interaction. These conserved interactions are of great interest because the mechanisms that transduce APP and/or PrP^C^ dysfunction are attractive as therapeutic targets in various neurodegenerative diseases. The conservation of the interaction, confirmed independently by co-immunoprecipitation of human APP and PrP^C^, implies a fundamental biological importance to the phenomenon.

## Results

### *appa*, *appb*, or *prp1* Knockdown in Zebrafish Produces Morphological Defects and CNS Cell Death

**Figure 1:**
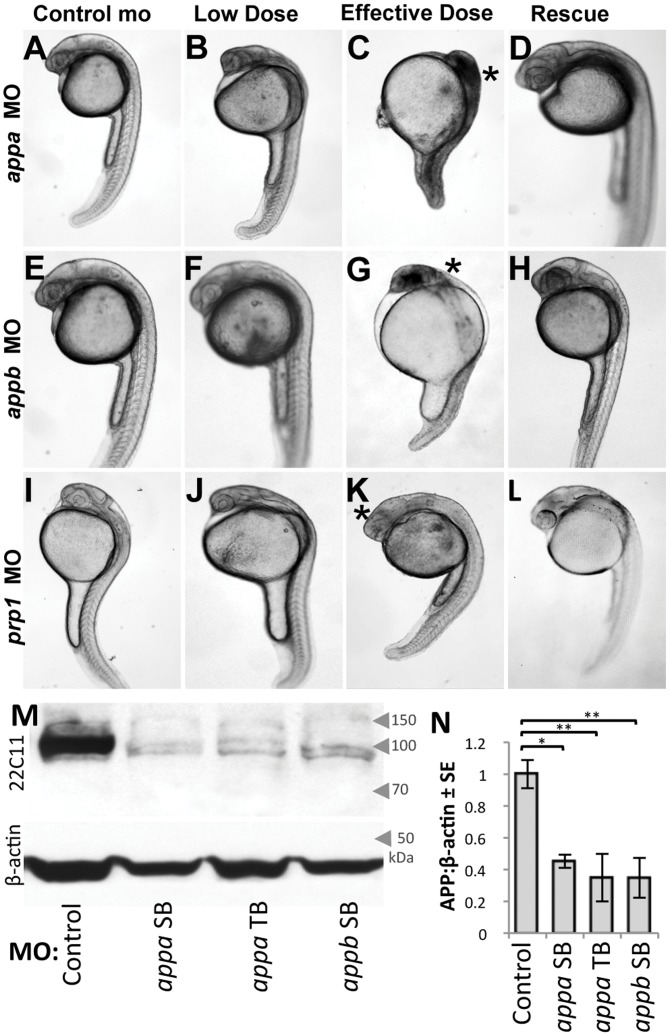
Knockdown of Appa, Appb, or Prp1 results in impaired development and death of head region. A–L. Morpholino (MO) was delivered to disrupt translation of endogenous amyloid β precursor protein (APP) and prion protein (PrP) paralogs in zebrafish: *appa*, *appb*, or *prp1* (top-bottom rows, respectively). Standard control MO at levels equivalent to our effective dose fail to induce any CNS cell death or disruptions in morphology of the fish (left column). Low doses of *appa, appb, or prp1* MOs (0.5, 1.0, 0.5 ng respectively) were empirically determined to be sub-effective (Fig. S1,S2), leading to mild changes, but no death of CNS tissues (2^nd^ column). Effective doses (1.0, 2.5, 1.0 ng, respectively) lead to severe alterations in CNS morphology (*) and death of CNS tissues (3^rd^ column). Specificity of the MOs was demonstrated by rescuing the injection of an effective dose of *appa, appb, or prp1* MO by co-injection of the cognate mRNA (200 pg, 200 pg or 100 pg, respectively; Right column). These data are quantified in Figs. 2F, S1 & S2. **M.** Western blots of zebrafish lysates reveal efficacy of our MO knockdown reagents (see also Fig. S3). The *appa* and *appb* splice blocking (SB) MOs used above (A–H) significantly decreases detection of protein species by the antibody 22C11 (top row). Bottom row is a β-actin loading control. An additional, independent MO that acts as a translation block of *appa* (*appa* TB) confirms this protein knockdown and produces similar phenotypes (Fig. S1). The *prp1* MO reagents used here were previously shown to be effective in knocking down protein [27]. **N.** Quantification of western blots from three biological replicates (three independent injection trials on three separate days) demonstrate a significant decrease (*p<0.05, **p<0.01) of the APP immunoreactivity compared to β-actin with all three MO reagents at their effective doses.

*appa* mRNA splicing was disrupted in wild type zebrafish by injecting a splice-blocking morpholino (MO) designed to bind the exon-intron boundary between exon 2 and intron 2–3. For *appb* the site chosen was the exon-intron boundary between exon 3 and intron 3–4. In both cases these targeted sites are upstream of highly conserved regions of the genes that are present in all splice isoforms. Delivery of *appa* or *appb* MO at high (effective) doses resulted in fish displaying a neurodevelopmental phenotype comprised of overt physiological malformations; cranial edema, reduced body size, improper CNS development and structure, and the presence of CNS cell death evident as dark necrotic-like regions disrupting transmission of light through the otherwise transparent embryo (Fig. 1C, 1G).

Morpholino *efficacy* was confirmed by multiple methods: (**i**) RT-PCR demonstrated altered mRNA splicing in the presence of MO, producing PCR products of a size that indicated retention of the expected MO-adjacent intron (Fig. S1, S2; the sizes of these PCR products allowed us to exclude a hypothetical contamination of genomic DNA in the RNA preparations, because our primers spanned multiple introns in the target gene). (**ii**) Sequencing of these RT-PCR products demonstrated that multiple termination codons are present in the altered transcript, predicting a truncated protein (Fig. S1). (**iii**) There was a dose-response relationship between the amount of injected MO and appearance of the phenotypes (Fig. S1, S2), and (**iv**) by quantifying protein knockdown from three biological replicates, representing delivery of all MO reagents on three separate days (Fig. 1M, S3). Protein levels were reduced, with a mean reduction amongst different trials being greater >50% (Fig. 1N, p<0.05, mean of three biological replicates. 22C11 detects both Appa and Appb, see Fig. S3).

**Figure 2:**
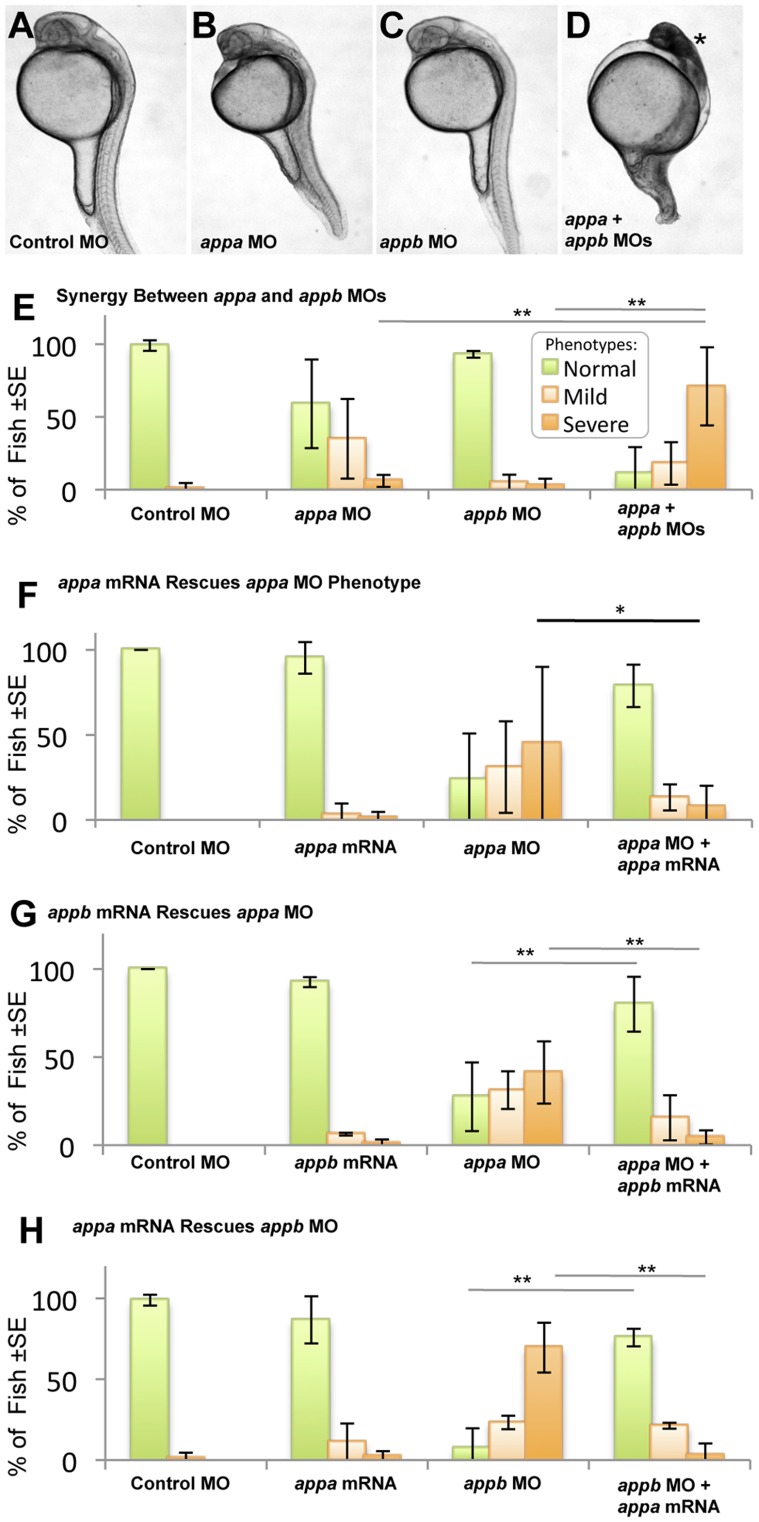
Appa and Appb can replace each other and thus are redundant in early zebrafish development. A–D . Embryos were injected with sub-effective doses of *appa* and/or *appb* morpholino (MO). These doses produced no phenotype in the fish compared to control MO (A–C, see also Figure 1). **D.** When sub-effective doses of *appa* and *appb* MO were combined and injected, a strong phenotype emerged consisting of morphological malformations and death of tissues within the CNS (*). **E.** Quantifying this effect, the co-injection of sub-effective doses of both MOs produced a significant decrease in the number of normal fish (green bars) and a significant increase in number of fish displaying CNS cell death (mild in light orange bars; severe in dark orange bars). ** = P<0.01. **F.** Fish injected with *appa* MO can be rescued by co-injection with *appa* mRNA * = P<0.05. A similar result was attained for *appb* MO and its cognate mRNA (Fig. S2). **G, H.** Fish were injected with an effective dose of one MO along with cognate mRNA from the other paralog to see if rescue of the phenotype occurred. *appb* mRNA was able to effectively alleviate the phenotype caused by injection of the *appa* MO (G) and vice versa (H). ** = P<0.01.

MO *specificity* was confirmed by (**i**) multiple trials quantifying the significant rescue of MO-induced phenotypes with MO-insensitive mRNA from the cognate gene (p<0.05 in each case, Fig. 1, 2f, S2, see also Table 1). The overall abundance of dead embryos throughout our trials was found to be 23±13% of the embryos examined and this value did not vary substantively between MO or mRNA injection treatments (Table 1); (**ii**) Failure to rescue the MO-induced phenotypes when these same mRNAs had termination codons engineered into them (Table 1, and see below); (**iii**) Failure to rescue phenotypes when the mRNA from related genes were delivered; (**iv**) Delivery of a second MO reagent designed to block *appa* translation by binding to a disparate portion of the gene, the 5′UTR, produced the same phenotypes (Fig. S1, see also Fig. 1M and S3 demonstrating efficacy of this *appa* translation blocking (TB) MO in reducing protein abundance). The quantity of MO and/or mRNA reagent delivered was kept consistent between trials *via* the calibration of injection volumes using an ocular micrometer. Overall these tests of reagent efficacy and specificity represent the quantification of phenotypes in several thousand individual fish involved in more than 100 separate trials (Table 1 and Protocol S1).

**Table 1:** Summary of phenotypes attained and number of trials per treatment applied.

MO/mRNA Combination^1^	Dose (ng MO, pg mRNA)	Phenotype observed (%)			N = # of trials	n = total # of fish	% dead ±SD	Figure
		normal	mild	severe				
Control MO	2.5	99	1	0	19	796	19±10	2e, g, h, 3e, 3j, S1k, S2d
Control MO	1	100	0	0	12	356	19±12	2f, g 3k
*appa* MO	2.5	24	31	45	3	151	20±07	2f
*appa* MO	0.5	67	24	9	19	578	22±13	2e–g, 3e
*appa* mRNA	200	91	7	2	6	172	24±12	2f,2h
*appa* MO+*appa* mRNA	2.5+200	79	13	8	3	151	19±07	2f
*appa* MO+*appa* mRNA	1.0+200	81	14	5	1	43	14	S5a
*appa* MO+*appb* mRNA	1.0+500	80	16	4	3	90	20±05	2g
*appa* MO+*appa*∧S3X;E5X mRNA	1.0+200	15	27	58	1	33	34	S5a
*appa* MO+*appa*∧14–15insT mRNA	1.0+200	26	48	26	1	35	30	S5a
*appb* MO	2.5	5	12	83	5	153	28±18	2h, S2d
*appb* MO	1	94	6	2	6	279	17±15	2e, 3j
*appb* mRNA	500	93	6	1	3	80	25±09	2g
*appb* MO+*appb* mRNA	2.5+200	82	14	5	3	117	17±02	S2d, S5b
*appb* MO+*appb*∧M3X;V7X mRNA	2.5+200	3	8	89	1	37	8	S5b
*appb* MO +*appb*∧del8A mRNA	2.5+200	3	3	94	1	34	15	S5b
*appb* MO+*appa* mRNA	2.5+200	76	21	3	3	66	27±06	2h
*appa* MO+*appb* MO	0.5+0.5	11	18	71	3	140	22±04	2e
*prp1* MO	0.5	95	4	0	17	503	22±12	3e, 3j, S1k
*appb* MO+*prp1* MO	1.0+0.5	84	16	0	3	133	11±08	3j
***appa*** ** MO+** ***prp1*** ** MO**								
*appa* MO+*prp1* MO	0.5+0.5	8	26	66	10	303	30±09	3e, 6a
+ *appa* mRNA	+200	67	27	7	4	132	26±13	3l, 3k, S5c
+ *appb* mRNA	+200	7	31	62	7	217	32±13	3k, 6b, S5c
+ *appa*∧S3X;E5X mRNA	+200	5	51	44	1	43	14	3L, S5c
+ *appa*∧14–15insT mRNA	+200	9	53	38	1	32	36	3L, S5c
+ *appb*∧M3X;V7X Mrna	+200	0	31	69	1	36	28	S5c
+ *appb*∧del8A mRNA	+200	10	35	55	1	31	38	S5c
+ human *APP* mRNA	+200	43	33	24	3	112	14±04	6b
+ *prp1* mRNA	+100	55	35	10	3	71	26±17	6a
+ *prp2* mRNA	+100	18	46	36	3	66	13±06	6a
+ *sho1* mRNA	+100	15	45	40	3	87	28±14	6a
+ mouse *Prnp* mRNA	+100	33	39	28	3	72	33±09	6a
**APPa TB-MO** 2								
*appa* TB-MO	2.0	30	40	30	1	27	39	S1k
*appa* TB-MO	1.0	85	15	0	1	27	35	S1k
*appa* TB-MO+*prp1* MO	1.0+0.5	29	42	29	1	38	34	S1k
**TOTAL**					**156**	**5241**	23±12	

1 Treatments were combinations of morpholino (MO, designed to block normal splicing) gene knockdown and/or mRNA gene expression reagents.

2 A translation blocking MO (TB-MO) was used as an independent knockdown reagent to validate some results.

We observed similar phenotypes as those above during knockdown of zebrafish *prp1* (Fig. 1K), using a translation blocking MO previously reported [27] to reduce Prp1 protein abundance. Fish injected with the low dose (0.5 ng) of *prp1* MO presented with a delay in development, some slight CNS malformations, and at higher doses (1 ng) began to show signs of apoptotic cell death (Fig. 1J, K). These results differ from those previously reported [27] insofar as usage of a lower dose of the MO knockdown reagent allowed us to examine effects of Prp1 knockdown at developmental stages beyond gastrulation.

Our attempts to establish *prp2* MO reagents met with some success, reproducing aspects of previous studies [27], [28]. However, these reagents failed to meet the most stringent tests of reagent specificity [29], insofar as we could not rescue the *prp2* knockdown phenotypes with *prp2* mRNA, consistent with previous reports [27]. Consequently we addressed the role of *prp2* in the interactions of interest by delivering it as an mRNA in the experiments below.

### *appa* and *appb* are Redundant in Early Development

The predicted Appa and Appb proteins share 70% sequence identity overall (>90% in several domains, Figure S4) and substantially overlap in gene expression domains [13]. To test the hypothesis that products of the *appa* and *appb* genes are redundant, we completed concerted knockdown and tested the ability of these paralogs to replace each other. Doses of MOs that disrupt the splicing of *appa* and *appb* were reduced to levels that produced little observable phenotype (“sub-effective” doses; 0.5 ng *appa*, 1.0 ng *appb*) (Fig. 2A–C, E). When these sub-effective doses of *appa* and *appb* MO reagents were co-injected there was a significant increase in the percentage of fish displaying a severe phenotype (Fig. 2D, E, Table 1), including both morphological malformations and CNS cell death. Messenger RNA from one paralog was shown to significantly rescue the phenotype caused by knockdown of the other paralog (P<0.05) (Fig. 2G, H). The quality of this gene replacement in rescuing the observed phenotypes was comparable to *appa* mRNA rescuing Appa knockdown (Fig. 2F, equivalent for *appb* presented in Fig. S2). Thus *appb* mRNA was able to effectively rescue the phenotype caused by a knockdown of the APPa protein, and *vice versa* (Fig. 2F, G). We conclude that, within the context of our assays, the paralogs *appa* and *appb* are formally redundant during the development of wild type zebrafish.

### *appa* Interacts Genetically with *prp1*

**Figure 3:**
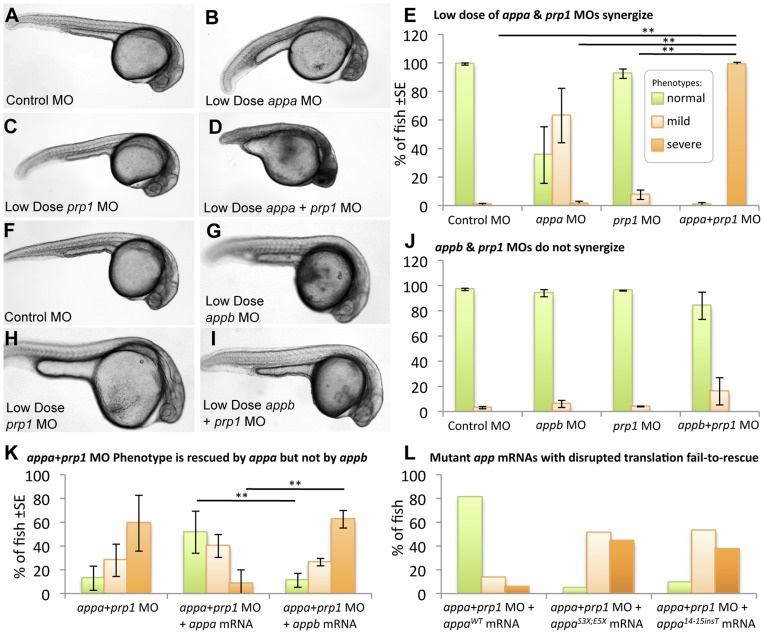
*appa* interacts with *prp1,* but *appb* does not. Panels **A–E**: Sub-effective doses of *appa* and *prp1* gene knockdown synergize to produce an overt phenotype in the fish. Fish injected with a control morpholino (MO) (**A**), a sub-effective dose of *appa* (**B**) or *prp1* (**C**) MO fail to display any signs of CNS cell death or disruptions in development, i.e. no severe phenotypes. **D.** When sub-effective doses of *appa* and *prp1* are combined a severe phenotype emerges comprised of prominent morphological disruptions and an overt appearance of cell death within the CNS. **E.** The abundance of fish with normal morphology observed is significantly reduced, and the percentage of fish displaying cell death within the CNS is significantly increased when sub-effective doses of *appa* and *prp1* MOs are combined. ** = P<0.01. Panels **F–J** present a similar experimental design to panels A–E, but represent *appb* knockdown instead of *appa*. When a sub-effective doses of *appb* and *prp1* MOs are combined there is no significant increase in the number of fish showing developmental abnormalities or cell death within the CNS. **K**. Despite Appa and Appb being largely redundant during normal development (Fig. 2), they cannot replace each other when PrP1 abundance is reduced. *appa* mRNA is able to alleviate the phenotype caused by co-injection of sub-effective doses of *appa* and *prp1* MOs. *appa* mRNA significantly reduced the percentage of fish displaying a severe phenotype. *appb* mRNA at an equivalent dose failed to reduce the percentage of fish displaying a phenotype. ** = P<0.01. **L.** *app* mRNAs with stop codon mutations are not able to rescue the *app* or *appa*+*prp1* knockdown phenotypes. Data from the mutations S3X;E5X and 14_15 insT are shown (WT = wild type). Further analysis of these mRNAs and similar ones for *appb* was carried out in other knockdown backgrounds (Fig. S5).

Based on the similarity between the Appa, Appb and Prp1 knockdown phenotypes (above) and prior reports in the literature indicating that murine APP and PrP^C^ proteins may physically interact [7]–[9] we tested for interactions between the zebrafish homologs of *APP* and *PRNP* genes. When sub-effective doses of the *appa* (0.5 ng) and *prp1* (0.5 ng) MOs were injected alone many fish displayed slight morphological malformations (63±38%, 8±7% respectively, Table 1), but only 2±3% displayed any signs of CNS cell death (Fig. 3A–E). When these knockdown reagents were co-injected there was a ∼50-fold increase in the percentage of fish displaying both peripheral and CNS malformations along with cell death, reaching a figure of 99±3% (P<0.01; Fig. 3E, Table 1). Specificity of this concerted Appa plus Prp1 co-knockdown phenotype was demonstrated by significant rescue of the phenotype by co-injection of *appa* mRNA (p<0.01, Fig. 3K). This overall conclusion that *appa* has a genetic interdependence with *prp1* was further confirmed with an independent *appa* MO designed to block translation (Fig. S1E–K).

### *appa* and *appb* are not Redundant when Prp1 is Reduced

Considering the redundancy of *appa* and *appb* demonstrated above during normal development (Fig. 2), a functional interaction study was also conducted with *prp1* and *appb*. Both the *prp1* MO and the *appb* MO, when injected at sub-effective levels, resulted in few fish displaying CNS malformations (4±1% and 6±5% respectively, Table 1), and no cell death within the CNS was observed (Fig. 3G, H, J). When these same sub-effective doses of *prp1* and *appb* MOs (0.5 ng *prp1* and 1.0 ng *appb,* respectively) were combined there was no significant change in the percentage of fish displaying CNS malformations (16±19%, Table 1), and no significant increase in the percentage of fish displaying CNS cell death was observed (Fig. 3F–J). Thus the hypothesis that *appb* has a genetic interaction with *prp1* is not supported, in sharp contrast to our observations regarding *prp1* and *appa*. We tested the alternate hypotheses that the genetic interaction of *prp1* with *appa*, and not with *appb*, was (i) an idiosyncrasy of our MO reagents affecting different exons, or (ii) a result of different spatiotemporal expression domains between *appa* and *appb*. Concerted injection of *appa* mRNA was able to rescue the Appa plus Prp1 knockdown phenotype (Fig. 3K, P<0.05); in contrast injection of *appb* mRNA was not (Fig. 3K). Thus expression of *appa* from mRNA, likely representing ectopic over-expression, was able to rescue the phenotype whereas *appb* mRNA was not (Fig. 3K), eliminating these alternate hypotheses. In sum, *appa* and *appb* are largely redundant during normal zebrafish development (see Results section above and Fig. 2), yet cannot replace each other during the development of the CNS in fish that have reduced levels of Prp1 protein. Thus the interactions of Prp with App we report cannot be an artifact of the methods we used, and instead represent a surprising paralog-specific niche interaction of Appa and Prp1 required for normal CNS development.

### Phenotypes Observed are Caused by Decreased Protein Abundance

Investigating the etiology of the MO-induced phenotypes was warranted in light of previous works reporting a lack of overt phenotypes during *appa* loss-of-function in zebrafish [24], [25]. We sought to more completely assess if decreases in Appa protein abundance, induced by the *appa* MO (Fig. 1M, N), cause the observed developmental deficits. We thus assessed alternate hypotheses that the *appa* mRNA we injected to rescue the phenotype was either (i) having a direct effect on the system by itself (perhaps akin to known interactions of PRP and SHADOO proteins with RNA species, or via interactions of *APP* mRNA with cytoplasmic proteins [30]–[35]) or (ii) it was required to be translated to Appa protein. To an extent this concept is tested above (Fig. 3K), wherein *appa* mRNA, but not *appb* mRNA, was able to rescue the phenotype induced by co-injecting *appa* plus *prp1* MOs. To directly appraise these hypotheses we engineered termination codons into the beginning of the *appa* mRNA (Fig. S5D) and compared its efficacy of rescue to wild type mRNA. Neither an *appa* mRNA with stop codons included near the 5′ of the transcript (*appa* ^S3X;E5X^), nor an *appa* mRNA with a single nucleotide insertion producing a frameshift (*appa* ^14_15 insT^), were able to rescue the concerted Appa plus Prp1 co-knockdown phenotype (Fig. 3L, S5C). These mutant mRNAs were also unable to rescue Appa knockdown (Fig. S5A), despite *appa* wild type mRNA having consistent efficacy in rescue experiments (e.g. Fig. 2F, S5A, Table 1). In sum, a failure to rescue our phenotype was noted when we subtly altered the *appa* mRNA at one or two nucleotides (representing less than 0.1% of the mRNA, see Fig. S5D) in two distinct approaches that eliminate translation of a functional protein. Thus we conclude that the *appa* mRNA must be translated to Appa protein to exert its effects on the concerted App plus Prp co-knockdown phenotype. We attained analogous results by altering the *appb* mRNA in two distinct ways (*appb* ^M3X;V7X^ or *appb* ^del8A^, Fig. S5D) and found that these mutations render the mRNA unable to rescue the Appb knockdown phenotype (Fig. S5B), consistent with our data showing decreased APP-immunoreactive protein in fish injected with *appb* MO (Figs. 1M, N and S3). The data are consistent with our MO reagents against *appa* being efficacious and specific. Past disruptions of this gene with a different MO were not directly tested for knockdown efficacy [24], and mutants with insertions in *appa* introns produce protein with partial function and continue to produce wild type proteins and thus are not null alleles [25]. Finally, we note that the interaction of *appa* with *prp1* was confirmed using a second MO against *appa* designed to work in an independent fashion (by blocking translation) and this independent MO showed the same phenotypes and knockdown of APP immunoreactivity (Figs. 1M, N and S1, S3).

An alternative mechanism through which the splice blocking MO reagents could induce phenotypes is by the creation of truncated *trans* dominant proteins. Some assurance against this interpretation is the lack of any detectable truncated protein using the antibody 22C11 as above (Figure S3; the antigenic site is expected to be 100% intact in truncated Appb and ∼75% intact in truncated Appa following injection of the respective splice blocking MOs), presumably due to the degradation of transcripts whose processing was altered by MOs via nonsense-mediated decay. Regardless, we tested this possibility by delivering mRNAs encoding the protein that might be predicted to be generated following injection of *appa* or *appb* MO, to directly test for dominant effects. Gene fragments were assembled to template synthesis of these mRNAs, and their delivery had no noticeable effect on the embryo survival or development (Figure S6). Inclusion of fluorescently labelled dextran in the mRNA injection solution, and subsequent fluorescent microscopy, provided assurance that these mRNAs were delivered to the embryo with good fidelity. In sum, truncated proteins are not detectable following injection of the splice blocking MOs, and any hypothetical dominant effects associated with such proteins has been ruled out, at least with respect to the phenotypes reported herein. The observation of phenotypes using translation blocking MO against *appa* is further important assurance against this alternate explanation for the observed phenotypes (Figs. 1M, N and S1, S3).

Overall, the MO reagents deployed induce a significant decrease in the abundance of their target proteins (Figure 1M, N and S3); The experiments described in this section eliminate alternate hypotheses, leaving the conclusion that decreases in protein abundance cause the phenotypes observed following delivery of the MOs.

### Appa, Appb, or Prp1 Knockdown in Zebrafish Leads to an Increased Activation of Caspase 3

**Figure 4:**
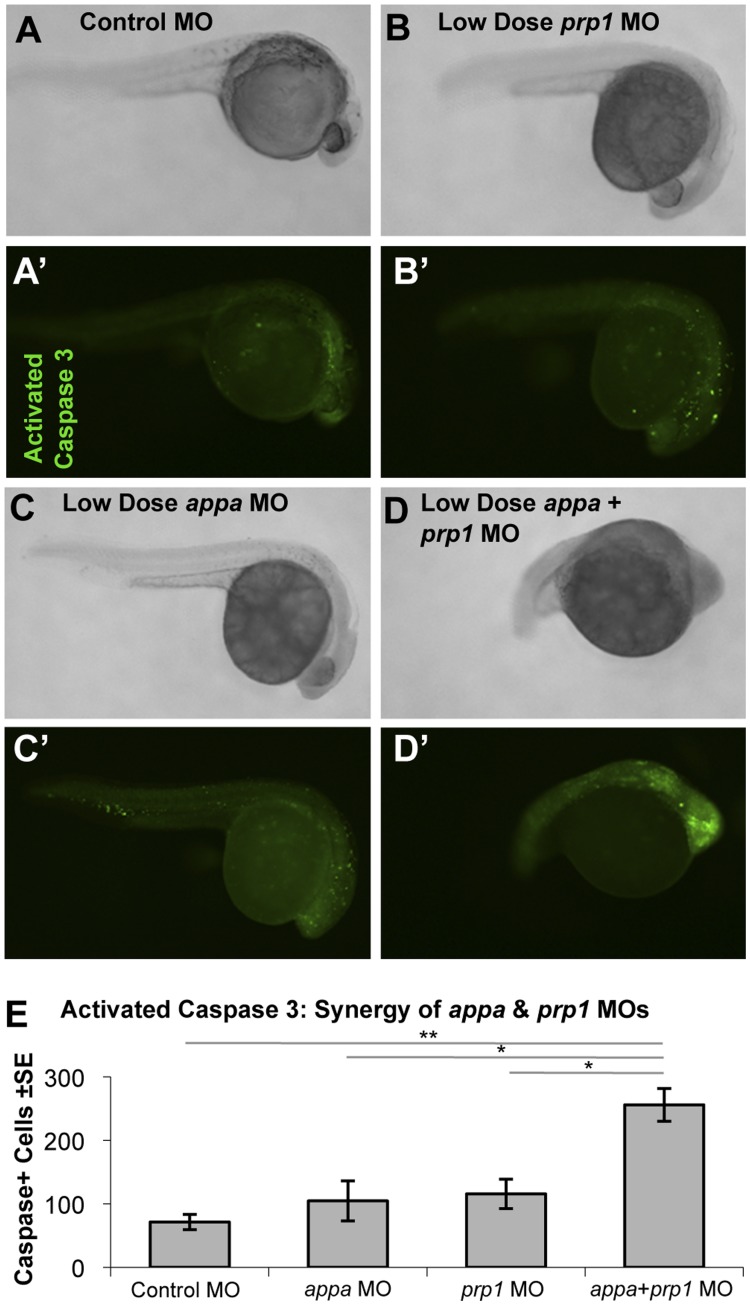
Apoptosis is synergistically increased when Appa and Prp1 levels are reduced. A–D. Zebrafish injected with a control morpholino (MO), low dose (sub-effective) *prp1* MO, low dose (sub-effective) *appa* MO, or a combination of sub-effective *appa* and *prp1* MOs (A–D, respectively) showed increased abundance of activated-caspase 3-positive cells (A′–D′, respectively). Higher doses of MOs used in this same assay showed individual MOs can also produce this effect (Fig. S7). **E.** Activated caspase 3-positive cells were slightly increased when low doses of *prp1* or *appa* MOs were injected alone and synergistically increased when they were combined in one injection solution. N = 5. ** = P<0.01, * = P<0.05.

APP and PRP have been hypothesized to have anti-apoptotic roles (reviewed in [15], [16]). The cell death we observed in fish injected with the *appa*, *appb*, or *prp1* MOs is consistent with this hypothesis (Fig. 1). To analyze this effect more closely, fish were fixed and stained with an antibody that detects a neoepitope formed by the proteolytic processing of caspase 3 to the enzymatically active form (Fig. 4, S7). Prominent labeling was apparent in both the periphery and the CNS, especially in the mid and forebrain regions, of fish injected with effective doses of *appa*, *appb*, or *prp1* MOs. To quantify the staining, fish were staged and the number of caspase 3 positive cells were documented, using the yolk sac extension landmark as per previous work [36]. Fish injected with the *appa* MO showed a significant (P<0.05) increase in the average number of activated-caspase 3-positive cells (135.2±33, N = 5) as did fish injected with the *appb* or *prp1* MOs (228±41 and 78±50 respectively, N = 5, P<0.05) when compared to control injected fish (23.2±10, N = 5) (Fig. S7). Apoptotic cell death was significantly (P<0.01) increased in fish injected with a combination of sub-effective doses of the *appa* and *prp1* MOs (Fig. 4).

### *appa* and *prp1* Interactions Modulate Cell Adhesion

**Figure 5:**
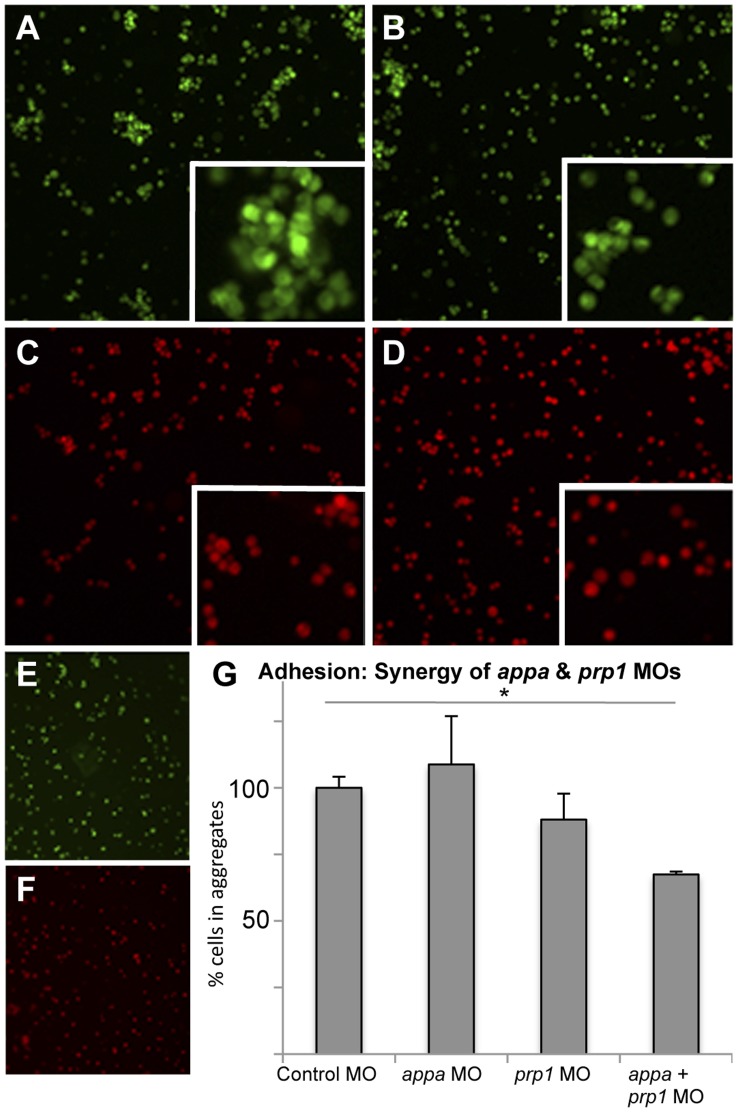
Knockdown of APP and PrP synergize to reduce cell aggregation. Low doses of *prp1* and *appa* knockdown reagents (morpholinos, MO) are used here to show that their effects on cell adhesion synergize; higher doses of MOs used in this same assay showed individual MOs can also produce this effect. **A–D.** Zebrafish embryos injected with fluorescent dyes along with control MO (A), low dose of *prp1* MO (B), low dose of *appa* MO (C), or a combination of the two sub-effective MOs (D), were dissociated to single cells and subjected to an aggregation assay. Insets show clumped cells (or lack thereof) at higher magnification. **E, F.** Aliquots of dissociated cells taken prior to aggregation confirmed that dissociation was successful. **G.** The ability of these cells to form aggregates (10 or more cells in direct physical contact) rather than stay alone in solution was quantified. Cells with slightly reduced App or slightly reduced Prp had only marginal decreases in aggregation ability, whereas cells with both proteins reduced were significantly reduced in their aggregation ability. N = 3. * = P<0.05.

To test the hypothesis that APP and PrP interactions play a role in cell adhesion, *appa* and *prp1* MOs were injected and the aggregating ability of cells was examined. Cells from embryos that had been injected with MO solutions and dextran dyes were first dissociated such that less than 10% remained aggregated (Fig. 5E, F: 92±4% disaggregated). These cells were incubated and the number of cells present in aggregates (10 or more cells in physical contact) was quantified by automated microscopy. Sub-effective knockdown of *appa* or *prp1* had no significant effect on the aggregating ability of cells (a 9% increase or 11% decrease respectively in aggregation compared to control MO), but when sub-effective doses were combined there was a 33% decrease in the number of cells in aggregates (Fig. 5G; P<0.05). Equivalent results were obtained in independent experiments quantified manually by a blinded observer rather than by automated image processing robot. Delivery of *appa* and *prp1* mRNA showed the converse effects, wherein they individually had no significant effect on cell aggregation (19% and 40% increases in aggregation compared to control mRNA, respectively). Combining these same doses of *appa* and *prp1* mRNAs had a tendency to increase cell aggregation, but did not reach significance (113% increase, p<0.181).

### *appa* Interacts with *prp1*, but not Other Prion-family Members

**Figure 6:**
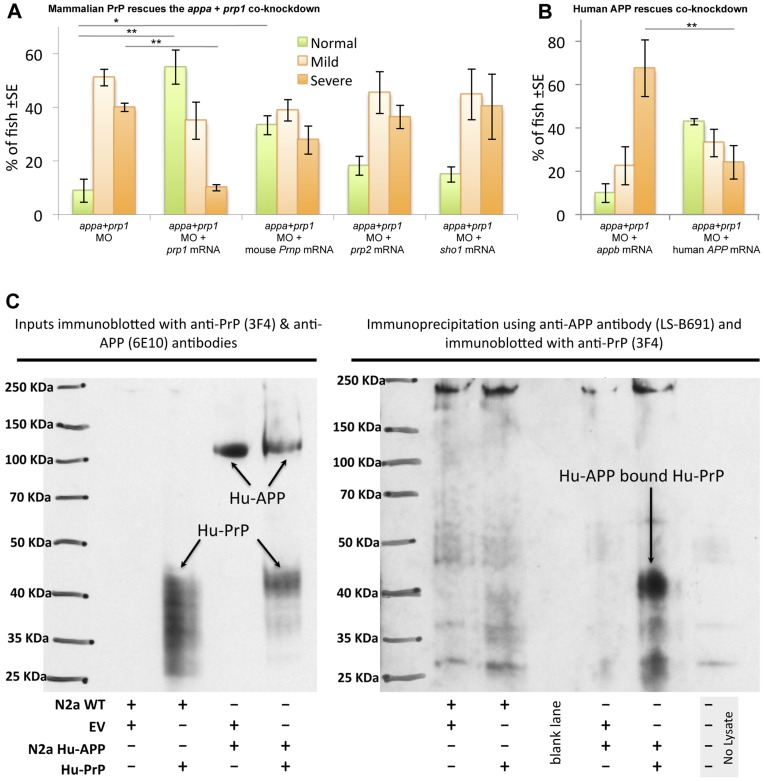
APP interactions with PrP are conserved from fish to mammals. A. Mouse *Prnp* can replace zebrafish *prp1* in the context of its genetic interaction with *appa*. Co-injecting zebrafish *prp1* mRNA, in concert with the Appa+Prp1 co-knockdown, rescues the observed phenotypes (first two sets of bars). *prp1*’s paralog, zebrafish *prp2*, does not rescue this co-knockdown, nor does another prion family member from zebrafish, *shadoo1*. In contrast, mouse *Prnp* mRNA (*moPrP*) can partially alleviate the Appa & Prp1 co-knockdown. Thus mouse PrP can replace Prp1 in the context of its interaction with App, indeed with greater efficacy than zebrafish orthologs. * p<0.05. **p<0.01. **B. Human** ***APP*** **can replace zebrafish** ***appa*** **in the context of its genetic interaction with** ***prp1*** **.** We established above that *appa* mRNA from zebrafish can rescue the co-knockdown of Appa+Prp1; Here we use *APPb* as a negative control comparator mRNA (see Fig. 3K). Human *APP_695_* mRNA (huAPPwt) was effective in replacing zebrafish APPa in the context of Prp1 knockdown. **C. Co-immunoprecipitation demonstrates an interaction between human PrP and human APP in N2a cells.** Left: Inputs as whole cell lysate showing expression of human PrP using the human PrP specific antibody 3F4 in N2a cells (wild type and stably transfected with human APP) transiently transfected with pcDNA3-human PrP construct but not with empty vector (“EV”). Expression of human APP is only observed in N2a cells with human APP using 6E10 antibody, specific for human APP. Input represented 7% of whole cell lysate used for co-immunoprecipitation. Right: whole cell lysates were co-immunoprecipitated using a human specific anti-APP antibody followed by immunoblotting with a human PrP specific antibody. Detection of human APP bound human PrP was observed only in N2a cells stably transfected with human APP and transiently transfected with human PrP construct. A no lysate immunoprecipitation experiment was included as an additional negative control.

Intrigued by *prp1*’s paralog-specific interactions with *appa* versus *appb*, we similarly tested genetic interactions in regards to PrP paralogs. Concerted mRNA injections, in a background of Appa plus Prp1 knockdown, demonstrated that 100 pg of *prp1* mRNA could rescue the phenotype (P<0.05) (Fig. 6A). In contrast, 100 pg of *prp2* or *sho1* mRNAs were not able to rescue the observed phenotype (Fig. 6A). Thus *appa* interacts with *prp1*, but not with the related prion family members *prp2* or *sho1*. Parallel to the conclusions derived from examining *APP* paralogs (Fig. 3K, L), these data are not consistent with our results deriving from idiosyncrasies of our approach. They indeed support the contention that the effects of our *prp1* MO are occurring via protein knockdown. This is consistent with past reports showing this *prp1* MO reduces Prp1 protein abundance [27].

**Table 2:** Primers used for gene cloning, checking morpholino efficacy and site-directed mutagenesis.

	Forward Primer (5′–>3′)	Reverse Primer (5′–>3′)
clone appa cDNA	AGAAGCATGCGGTCGAGGGA	GTGACGGTGCTCCATCAGTTG
clone appb cDNA	CAGCCATGGGTATAGACCGCA	TTAGTTCTGCATTTGCTCAAAGA
clone prp1 cDNA	CAAAATGGGGGAGTTATGCAAAC	CATTAAGTGGTACTAAAAAGCATAG
clone prp2 cDNA	ATGGGTCGCTTAACAATACTATTG	TGAGAATGTCAGTGTAGAAGGGA
clone sho1 cDNA	ATCCAGAATGAACAGGGCAGTC	CTCAAGGGGCAAAGTGCATCAT
Confirm appa MO efficacy	GAGCTCGAGGATGAACACTA	ACAGCGGCGCTCTCAGACT
Confirm appb MO efficacy	AGCCTGTCAGCATCCAGAAC	CACCGTCTTCATCGTTGTCC
Create appa∧S3X;E5X	CTAGAAGCATGCGGTAGAGGTAGCTCTTCATATTAC	GTAATATGAAGAGCTACCTCTACCGCATGCTTCTAG
Create appa∧14_15insT	CATGCGGTCGAGGGATGCTCTTCATATTAC	GTAATATGAAGAGCATCCCTCGACCGCATG
Create appb∧M3X;V7X	GCTCAGCCATGGGTTAAGACCGCACG TGATTCCTGCTTTTAATG	CATTAAAAGCAGGAATCACGTGCGGTCT TAACCCATGGCTGAGC
Create appb∧del7A	GCTCAGCCATGGGTTAGACCGCACGG	CCGTGCGGTCTAACCCATGGCTGAGC

### The Collaborative Roles of App and Prp can be Performed by their Mammalian Homologues

Based on the high conservation of domains present between zebrafish Prp and mouse PrP, we tested the hypothesis that mammalian PrP could replace zebrafish *prp1* in its interaction with *appa*. We found that 100 pg of mouse *Prnp* mRNA was able to rescue the phenotypes observed during concerted delivery of *appa* plus *prp1* MOs, and while this was not to the same extent as zebrafish *prp1* mRNA, it was significantly more efficacious than zebrafish *prp2* in this role (p<0.05, Fig. 6A).

Human APP shows similarity to the predicted zebrafish Appa protein (∼70% identity, Fig. S4) and, as such, rescue experiments were carried out using mRNA encoding human APP_695_. Human *APP* mRNA was able to efficiently (P<0.05) rescue the phenotypes caused by concerted delivery of *appa* plus *prp1* MOs (Fig. 6B) including the abundance of activated caspase 3 labeling (Fig. S7). To better visualize the CNS following *appa* and *prp1* knockdown and rescue, transgenic zebrafish *Tg(gfap:GFP)* (green fluorescence protein driven by the glial fibrillary acidic protein promoter) were employed (Fig. S7). Under fluorescence it was noted that when sub-effective doses of MO were co-injected there was reduced GFP expression in regions of the zebrafish CNS. Consistent with results above (Fig. 3K) we further noted that zebrafish *appb* mRNA was not able to rescue the concerted *prp1* plus *appa* knockdown; However human *APP_695_* mRNA was effective in restoring normal development (Fig. S7). Control MO injections confirm that the *Tg(gfap:GFP)* transgenic fish harbored no intrinsic susceptibility to MO injection as these fish developed normally (Fig. S7).

### Human APP and PrP Physically Interact in Mammalian Cells

The genetic interaction demonstrated above suggests that *APP* and *PRNP* might affect the same pathway(s), and this could be through a direct interaction of proteins or their common occurrence within a scaffolded protein complex. Alternatively, they may independently affect a downstream pathway; these hypotheses are not mutually exclusive. Past work describing the APP interactome identified PrP^C^ as one of the hundreds of proteins hypothesized to interact with APP in mice, and similar work suggests APP as a hypothetical member of PrP^C^’s interactome [7]–[9]. This data, combined with the impressive conservation of APP between zebrafish and humans (*e.g.* they can replace each other during development, see Fig. 6) led us to test the hypothesis that a biochemical interaction occurs between human APP holoprotein and human PrP. We performed co-immunoprecipitation from mouse neuroblastoma N2a cells stably or transiently transfected with human APP and human PrP, respectively. Detection of human APP bound in a complex with human PrP^C^ was observed only in N2a cells that were transfected with both human APP and with human PrP^C^ (Fig. 6C). Specificity of the reagents was confirmed by the lack of signal when either human APP or human PrP was absent from the cells. Thus APP holoprotein and PrP^C^ protein interact, and combined with the genetic interaction we show in zebrafish, we conclude this interaction is deeply conserved in vertebrates.

## Discussion

We sought to validate and expand upon putative APP-PrP interactions through an independent method. Our concerted *in vivo* knockdown of *APP* and *PRNP* homologues, combined with an mRNA replacement strategy, reveal that *APP* and *PRNP* homologues have a genetic interdependence in zebrafish. Our control experiments use mRNA to replace the cognate disrupted gene, and knockdown specificity is further verified by the limitation of the interactions to only one pair of *app* and *prp* paralogs (*appa* genetically interacts with *prp1*, but not *prp2*; *prp1* interacts with *appa* but not *appb*). Considering the immunoprecipitation of mammalian homologs (see also discussion of relationships in humans and rodents below), we interpret this to mean that APP and PrP interactions are highly conserved through at least 450 million years of evolutionary time, and thus important and worthy of further detailed study. The interaction we report, underpinning cell adhesion and CNS apoptosis, appears to be entirely relevant to mammalian orthologues in-so-much that human APP and murine PrP can replace their zebrafish orthologues during the APP-PrP interaction in our neurodevelopmental assays. The conservation is further underlined by our findings that (i) *appa* and *appb* can replace each other during normal zebrafish development, *i.e.* they are redundant in our assays; (ii) *appb* cannot replace *appa* during CNS development if PrP protein levels are reduced; (iii) in a surprising contrast to the latter, human *APP* mRNA is able to replace zebrafish *appa* during development of CNS with reduced PrP levels; (iv) similar to the latter point, in a context of reduced Appa levels, mouse *Prnp* is better able to replace zebrafish *prp1* compared to related zebrafish *prp2* or zebrafish *shadoo*.

From this we derive four important points: First, the MO knockdown and mRNA overexpression reagents we describe are all efficacious and specific to the extent that these results cannot be explained as being an idiosyncrasy of our interventions. Second, the interaction of *appa* with *prp1* is a specialized niche event during neurodevelopment, disruption of which has substantive consequences on cell adhesion and neuron survival. Third, the ability of human APP (but not zebrafish Appb) to replace zebrafish *appa* in the context of reduced PrP protein argues strongly that human APP interacts with PrP in a conserved neuroprotective role. We validated just such an interaction of human APP with human PrP using co-immunoprecipitations. Finally, this data sets the stage for comparisons amongst APP proteins to define which residues and domains are critical for the interactions with PrP and the effects on cell adhesion and neuron survival. We are actively pursuing the latter by investigating residues that are shared by human APP and zebrafish Appa but that are not shared by zebrafish Appb. Appa and human APP are more similar to each other than to Appb in only one contiguous location greater than three amino acids - the amino-terminal end of Aβ, i.e. the site of β secretase (BACE) cleavage (Fig. S4). The impressive conservation of the APP intracellular domain between human and zebrafish paralogs suggests that a conserved function of APP includes endoproteolytic cleavage towards intracellular signalling [37], and disruption of this processing via differences of BACE cleavage would then be expected to be consequential, consistent with a role for PrP^C^ in modulating BACE function [38].

### Potential Relevance to Disease

Alzheimer Disease and prion diseases represent insidious, slow and inevitably fatal neurodegenerative diseases. Myriad similarities exist between their endpoints and histopathologies, and their antemortem differential diagnoses remains challenging [39], [40]. Both diseases present as sporadic and familial forms, whereas prion diseases are differentiated from Alzheimer Disease in that they can also present as infectious forms. Pathologically, Alzheimer and prion disease share hallmarks of disease progression: short toxic protein oligomers that form into extracellular plaques containing both PrP^Sc^ and Aβ, early loss of dendritic spines and synaptic plasticity associated with learning deficits, tau hyperphosphorylation and neurofibrillary tangles, dysfunction in metal homeostasis, gliosis, neuronal apoptosis and dementia [41]–[44]. As expected from these similarities, differential gene expression points to overlaps in Alzheimer and prion disease endpoints [45], [46].

Our study represents an attempt to uncover putative genetic and biochemical relationships between disease effectors. It does not necessarily follow that our approach can uncover linkages between the diseases themselves, though we suggest that any such relationships ought to be relevant to early neuropathological progression, before the endstage commonalities described above. Our interventions herein primarily focus on loss-of-function approaches to study APP and PrP, and thus we must note that toxic gain-of-function, not addressed experimentally herein, is a driving force in both diseases. Indeed reduced levels of PrP or APP can ameliorate disease progression in some models [3], [47]. It is equally noteworthy that loss-of-function in APP and PrP are broadly accepted as playing substantive roles in their respective disease progressions [48]–[55]. Topical examples reviewed below include speculation that disrupting PrP^C^’s function leads to deregulation of Aβ production, and disruption of either APP or PrP^C^ can lead to deficits in synaptogenesis, neuroprotection and/or learning, perhaps through loss of their regulatory role upon metal homeostasis with special relevance to NMDA receptor-mediated plasticity. Further, recent findings suggest a complex integration of neuroprotective effects of PrP^C^ and the toxic effects of PrP^Sc^ [56], [57], such that causation during toxic PrP gain-of-function appear to be inseparable from the loss of neuroprotection. Our findings create a tractable paradigm for discovery of mechanisms whereby PrP loss-of-function leads to cell death, dependant on APP, and *vice versa*, and this may assist in resolving this debate. Regardless of the true balance, or entanglement, between loss- vs. gain-of-function at various stages of each disease’s etiology, it is apparent that interactions between APP and PrP have bearing on each disease.

In the recent past, the onset of Alzheimer and prion diseases were viewed as fundamentally different entities, though prescient speculations argued in favor of searching for etiological commonalities [39]. We briefly summarize the linkages between Alzheimer and prion diseases into three lines of evidence below, two of which remain controversial and the third being solidified for the first time by the co-immunoprecipitation data we report herein. The data in the current manuscript speak to a highly conserved and thus important interaction between PrP^C^ and APP, affecting cell adhesion and neuron survival, which we interpret as support for these biochemical and/or genetic interaction nodes.

Firstly, a vibrant literature suggests that PrP^C^ can act as a receptor for oligomerized Aβ, the disease-associated cleavage product of APP. The result of such binding was argued to influence synaptic plasticity and perhaps excitotoxicity [4]–[6], [58]–[63]. Indeed both PrP^C^ and Aβ may interact to mediate toxicity via regulation of NMDA receptors [64], [65] and/or K_V_ Channels [66]. The data sets from these groups are contentious regarding the effects of Aβ binding to PrP^C^ on long term potentiation and learning [4], [5], [58]–[63], though several groups have used a battery of techniques to repeatedly confirm high affinity binding of PrP^C^ to Aβ oligomers.

Secondly, human genetics has frequently, though inconsistently, described a controversial association of the human *Prnp* locus with risk for Alzheimer Disease. In particular, the PrP^M129V^ genotype that is protective in various prion diseases in a heterozygous state has been found to be associated significantly with Alzheimer Disease in several past and recent studies [67]–[70]. This association is not supported in all populations, which may be understandable in light of a multigenic risk factor for a late onset disease. Mechanistically, it has been shown that PrP^M129V^ polymorphisms modulate BACE (β secretase) cleavage of APP and thus affect levels of Aβ_42_ associated with increased Alzheimer Disease risk [38], [71]–[73]. Further, a well-documented Alzheimer Disease risk-associated locus, APOE-ε4, has also been shown to be linked with risk for sporadic prion diseases, though delayed onset of inherited forms of prion diseases in humans with Prnp^P102L^ is also observed [74]. P102L is within the region where Aβ oligomers bind PrP^C^ [6]. Thus human genetics tentatively suggests linkages between these diseases.

Finally, systems biology approaches have prompted the hypothesis that APP and PrP interact biochemically *in vivo*, though it cannot yet be excluded that intermediary binding partners are required. Protein interactomes of APP and PrP^C^ each independently annotate high-quality data that make APP and PrP^C^ likely interactors in rodent brains and cell culture paradigms [9], [75], [76]. This is consistent with APP and PrP^C^, representing Type I transmembrane and GPI-anchored proteins respectively, both being localized to the external leaf of cell membranes, at synapses and within lipid rafts [10]–[12]. Our co-immunoprecipitation studies validate the conclusions reached by these large-scale interactome studies, and, importantly, extend the conclusion to include human APP and PrP. These independent interactome studies also identify several protein interactors that APP and PrP^C^ have in common, including APLP1, neural cell adhesion molecule 1, integrins, and contactins, supporting the validity of the biochemical interaction and a common role for APP and PrP^C^ proteins in modulating cell adhesion [19]–[22]. Overall, then, identifying functional interactions between APP and PrP has substantial and diverse implications for Alzheimer Disease and prion disease research. Our data supports continued investigation of such linkages and establishes a tractable *in vivo* paradigm for their investigation.

### Conclusion

In sum, our comparison between APP and PrP paralogs has identified a conserved and specific niche role for APP-PrP interactions that are required for vertebrate CNS development, and the effects of disrupting this interaction are not the result of generalized decrements in neurodevelopmental integrity but are required for cell adhesion events. Considering the well-established role for APP and PrP (and cell adhesion proteins in general) in synaptic plasticity we speculate upon a role for APP-PrP interactions in modulating learning and/or excitotoxicity.

We conclude that APP and PrP have an important interaction affecting cell adhesion and neuron survival. This expands considerably on a recent flurry of work examining PrP^C^’s high-affinity binding of an APP catabolite, *i.e.* oligomerized Aβ (e.g. Refs [4]–[6]). The parent protein APP and its catabolite Aβ (and its oligomers) are substantially different entities, in-so-much that one is cell-membrane-embedded and able to initiate nuclear signalling & cell adhesion, whereas Aβ oligomers are primarily in the extracellular milieu, disease-associated and prone to aggregation into plaques in both healthy and Alzheimer diseased brains. APP normally exists as transmembrane dimers (or heterocomplexes), and its dimerization status affects Aβ_40/42_ ratios [77]–[79]. Thus an interaction of APP with PrP has the potential to be consequential at several nodes of Alzheimer Disease etiology. It is of interest to speculate that the high-affinity binding of PrP for Aβ is a consequence of PrP’s ancient and conserved interaction with the APP holoprotein. It may well be that Aβ disrupts this conserved interaction, and that understanding all the components of APP that biochemically bind PrP will inspire APP mimetics to interfere with PrP’s high-affinity binding to Aβ oligomers.

## Materials and Methods

### Ethics Statement

All zebrafish husbandry and experimentation were done under a protocol approved by the University of Alberta Animal Care and Use Committee under the auspices of the Canadian Council on Animal Care.

### mRNA Rescue Experiments

*appa*, *appb*, *prp1, prp2* and *sho1* cDNAs (Accession numbers JQ994487–JQ994491 associated with ZFIN numbers ZDB-GENE-000616-13, ZDB-GENE-020220-1, ZDB-GENE-041221-2, ZDB-GENE-041221-3, ZDB-GENE-031110-1) were cloned from wild type zebrafish into a PCS2+ or pCR2.1TOPO vector (primers in Table 2), and sequenced to confirm identity. mRNA for rescue experiments was synthesized using these plasmids, or variations thereof, as templates (see Protocol S1).

### Morpholino Injections

Six antisense morpholino oligonucleotides (MO) purchased from Gene Tools, LLC (Philomath, OR) were used during these experiments. These MOs were all designed to not bind the cognate mRNA constructs we produced, thus enabling rescue experiments. A splice-blocking morpholino was designed to specifically bind the exon-intron boundary of exon 2-intron 2 of the zebrafish *appa* pre-processed mRNA (APPa_SB 5′ TAG TGT TGC TTC ACC TCC TGG CAG T 3′). A translation blocking MO to the 5′UTR of *appa* designated APPa_TB (5′ GCT TCT GCT CCT CTT TAT TTC GCC T 3′). A splice-blocking morpholino designed to specifically bind the exon-intron boundary of exon 3-intron 3 of the zebrafish *appb* mRNA (5′ CAC ACA CAT ACA TAC CCA GGC AAC G 3′), and a previously published [5] translation blocking morpholino designed to specifically bind the 5′ UTR of zebrafish *prp1* mRNA (5′ TGA GCA GAG AGT GCT GCG GGA GAG A 3′). A standard negative control morpholino was obtained from Gene Tools (5′ CCT CTT ACC TCA GTT ACA ATT TAT A 3′). All morpholino injection solutions also contained a standardized dose (3 ng) of *tp53* morpholino (5′ GCG CCA TTG CTT TGC AAG AAT TG 3′; ZDB-MRPHLNO-070126-7 [80]) to counteract off-target effects of morpholino injection. Injection solutions were made using 1.0 µL of 0.1M KCl, 2.5 µL of 0.25% Phenol red, 1.2 µL of 25 mg/mL p53 MO stock, and gene-specific morpholino to effective (10 µg for *appa*, 10 µg for *prp1*, 25 µg for *appb*) or sub-effective (5 µg for *appa*, 5 µg for *prp1*, 10 µg for *appb*) concentrations, mRNAs as appropriate, and nuclease-free water to 10 µL. One cell stage embryos were staged on agarose plates, and injection volume calibrated to 1 nL using an ocular micrometer immediately prior to injection. Zebrafish injected with MOs and control MOs were staged at 24 hours post-fertilization (hpf) based on body morphology and screened for the presence of CNS cell death. Observer was blinded to treatment groups during screening of all phenotypes.

### Animal Husbandry

Zebrafish were maintained at 28.5°C in standard conditions [81]. Wild type (AB) strains were used for all experiments with the exception of rescue experiments using human APPs in which GFAP:GFP transgenic fish *Tg(gfap:GFP)mi2001* [82] were also used.

## Supporting Information

Figure S1 ***appa*** **morpholino (MO) injection leads to a dose-dependent disruption in** ***appa*** **mRNA processing. A.** Zebrafish embryos were injected with increasing doses of *appa* MO and scored for malformations and CNS cell death. **B.** Same experiments as panel A revealed doses that were toxic to the developing fish. **C.** *appa* splice block MO is efficacious, as it leads to disruption of *appa* mRNA. RNA was isolated from fish injected with *appa* MO, an equivalent dose of control MO, and subjected to RT-PCR. Fish injected with *appa* MO show a band at ∼300 bp corresponding to mRNA with intron 2 retained. This band is absent in when fish are injected with the control MO, or when standard Taq is used in place of reverse-transcriptase. **D.** Sequencing of the aforementioned ∼300 bp band confirms the retention of intron 2–3 in mature mRNA, and confirms our MO produces a truncated protein. Our sequence (top) was an exact match to zebrafish genomic clone NW_003336735 (bottom). Translation of the sequence, immediately 5′ of *appa* exon 3 (annotated in yellow at bottom right), predicts two termination codons (black, *). **E–J.** **A second MO reagent against** ***appa*** **was used to test specificity of the phenotypes observed.** Designed against a disparate portion of the gene, the 5′UTR and thus is a translation blocking (TB) MO. The efficacy of this MO is demonstrated in Fig. 1M and 1N. It produced mild and severe phenotypes (G and G′) indistinguishable from the splice blocking *appa* MO we primarily use in this work. The low dose of *appa* TB MO also showed a genetic interaction with the low dose of *prp1* MO, equivalent to results in Fig. 3 (J, J′). MO dose is indicated on panels. **K.** Quantification of *appa*-TB MO demonstrates a dose-dependant effect by itself and an additive effect with *prp1* MO during concerted delivery at sub-effective doses. Colour coding in the histogram is as per Fig. 2.
(TIF)

Figure S2 ***appb*** **morpholino (MO) injection leads to a dose-dependent disruption in** ***appb*** **mRNA processing. A.** Zebrafish embryos were injected with increasing doses of *appb* MO and screened based on presence of morphological malformations and CNS cell death. **B.** The same experiments as in panel A revealed doses that were toxic to the developing fish. **C.** *appb* splice block MO is efficacious, as it leads to disruption of *appb* mRNA. RNA was isolated from fish injected with 2.5 ng *appb* MO, 1.0 ng *appb* MO, or an equivalent dose of control MO, and subjected to RT-PCR. Fish injected with 2.5 ng of the *appb* MO show a band at ∼300 bp corresponding to retention of intron 3–4 in mRNA. This band is reduced when the dose of the MO is reduced, and absent when fish are injected with the control MO. Sequencing of the band confirmed the retention of intron 3–4 in mature mRNA, and predicted STOP codons in the modified mRNA. **D.** Embryos injected with the *appb* MO alone or with 200 pg of cognate *appb* mRNA. The instance of fish displaying a severe phenotype was significantly reduced and the number of normal fish was significantly increased upon inclusion of *appb* mRNA. **p<0.01. Colour coding in the histogram D is as per Fig. 2 and Fig. S1.
(TIF)

Figure S3 **Efficacy of** ***appa*** **and** ***appb*** **MO’s assessed by western blot.** MO knockdown reagents were assessed by Western blot to quantify protein abundance. Parts of this data appear in Fig. 1M. A. The size of the APP-immunoreactive bands in wild type mouse brain or from TgCRND8 mouse brains overexpressing human APP as detected with the antibody 22C11. B. Zebrafish App proteins are detected with 22C11 and the bands are indistinguishable from mammalian APP observed in panel A. Knockdown of zebrafish appa or appb gene products with various MO reagents (See Fig. 1, doses in nanograms are presented in brackets at the top of the figure) results in a significant reduction of APP immunoreactivity as normalized to β-actin levels and compared to control MO-injected fish. Smaller protein products that might be predicted to have a dominant effect following injection of splice blocking MOs are not detectable (predicted size of MO-altered proteins are 10 and 20 kDA for Appa and Appb, respectively). C. The 22C11 epitope (highlighted in blue) is conserved between human (top line) and zebrafish (Zf) proteins Appa (16/16 residues identical) and Appb (14/16 residues identical, 15/16 residues with conserved identity). Identity of the region is represented on the graph above the alignment with green showing perfect identity and amber showing mismatches.
(TIF)

Figure S4 **Conservation of App between zebrafish paralogs and human APP.** Amyloid β Precursor Protein (APP) is processed to Aβ (red), the major protein constituent of plaques in AD, by sequential enzyme cleavage. Zebrafish have two gene paralogues, *appa* & *appb*, wherein most residues of C99 at least one of them is a perfect match to human. Human APP is able to replace Appa in this interaction, Appa is more similar to human (purple boxes, red boxes show where Appb is more similar). Residues responsible for familial AD mutations (pink) are conserved in all three proteins.
(TIF)

Figure S5 ***appa*** **and** ***appb*** **mRNAs that rescue observed phenotypes must be translated to have their effect.** The phenotypes produced by knockdown of *appa* or *appb* can be rescued by co-injection of wild type *appa* or *appb* mRNA, respectively (Fig. 1–2, S1–S2, and left-hand data set of panels **A** & **B** here), but not by mutant mRNAs. **A, B. Assessment of mutant mRNAs.** Mutant mRNAs possessing point mutations in the start of the coding region fail to rescue phenotypes that are rescued by wildtype (WT) mRNA. **C.** The same result is found regarding the ability (or lack thereof) of these mRNAs to rescue the joint knockdown of *prp1* and *appa (*part of Fig. 2L is replicated here for clarity). **D.** Two separate alterations to each of *appa* and *appb* were made, with a goal of making subtle alterations to the mRNA molecule (<5 basepairs changed in the ∼2200 bp molecule) that ablate capacity to encode a functional full-length protein. In *appa*, we changed two basepairs (highlighted) at the start of the CDS that are predicted to create large changes in the protein by creating stop codons (S3X;E5X) (asterisks in protein represent stop codons) inducing a truncation. Alternatively, we modified the *appa* CDS by a single basepair insertion (14_15 insT) inducing a frameshift of 72 residues (grey shading) before a premature stop codon, and with only one of 72 residues having sequence identity with the parental wt mRNA. Similarly in *appb*, changing four base pairs created stop codons (M3X;V7X) and a truncated protein. We altered the *appb* mRNA in a second way, inducing a single basepair deletion (del8A) to create stop codons and a truncated protein. Stop codons are represented by asterisks, and residues N-terminal to stop codons are emphasized with strikethrough. Colour coding of histograms as per Fig. 2.
(TIF)

Figure S6 **mRNAs envisaged following injection of splice blocking morpholinos do not have a dominant effect.** Injection of the splice blocking MOs leads to inappropriate retention of the adjacent intron (with STOP codons); this is predicted to encode a truncated protein along with a portion of the retained intron. This altered mRNA may be degraded, but the kinetics are unknown so potential dominant effects of the predicted protein were tested, by delivering an mRNA encoding the truncated mRNA with retained intron. **A.** Delivery of mRNA encoding the first two exons of *appa* plus intron 2 (*appa-i2*, N = 3 trials, n = 114 fish). **B.** Delivery of mRNA encoding the first three exons of *appb* plus intron 3 (*appb-i3*, N = 2 trials, n = 91 fish). Data obtained from injecting mRNAs encoding the full length *appa* and *appb* proteins are presented for ease of comparison, replicated from Figure 2E and F, respectively. No dominant effect or significant change in phenotypes was observed following injection of *appa-i2* or *appb-i3* mRNA compared to injecting the cognate full length mRNA, or compared to injecting control MOs, or compared to uninjected fish. Thus MO injection likely leads to the phenotypes observed through reduction of protein abundance, and is not due to dominant effects of products from mis-spliced mRNAs. Colour coding of histograms as per Fig. 2.
(TIF)

Figure S7 **High doses of MOs affect apoptotic cell death, and Human APP rescues apoptotic cell death.** High doses of *prp1*, *appa* or *appb* MO are used here to show their individual effects on apoptotic cell death. Some combinations of these MOs co-injected at low doses show that these MOs can synergize to produce this effect (Fig. 4). Apoptosis levels are increased when *appa*, *appb*, or *prp1* mRNA is disrupted (**A–D**). Brightfield images of the area above the yolk sac extension of fish injected with effective doses of control, *appb*, *appa* and *prp1* (**A–D**, respectively) MOs. Compared with control fish, prominent anti-activated caspase 3 staining is apparent in *appb*, *appa*, or *prp1* MO injected fish (**A′–D′**, respectively). **E**. Number of caspase 3 positive cells were counted above the yolk sac extension in fish treated as per those in A–D. N = 5. Panels **F–K** show examples of human APP rescuing the concerted *appa* plus *prp1* knockdown. *appb* is not able to rescue the phenotype in wildtype fish, nor in transgenic fish labelling the CNS with GFP (H & I, respectively, compare to F & G) as we noted in Fig. 3K and here serves as a negative control. A noticeable lack of GFP was apparent along portions of the CNS (* in panel I). Human *APP* is able to rescue these phenotypes (**J**, **K**). Panels **L–N** show the yolk-sac extension of fish in F, H & J. L′–N′ show examples of activated caspase labelling during rescue with human *APP*. The latter treatments were quantified in **O**. N = 5. * = P<0.05. ** = P<0.01.
(TIF)

Protocol S1 **Supplemental description of Methods.**
(DOCX)
