# Supplementary figures and images for: Amyloid Beta Precursor Protein and Prion Protein Have a Conserved Interaction Affecting Cell Adhesion and CNS Development
Source: PLoS One. 2012 Dec 7;7(12):e51305. doi: 10.1371/journal.pone.0051305 (PMC3517466; doi:10.1371/journal.pone.0051305)

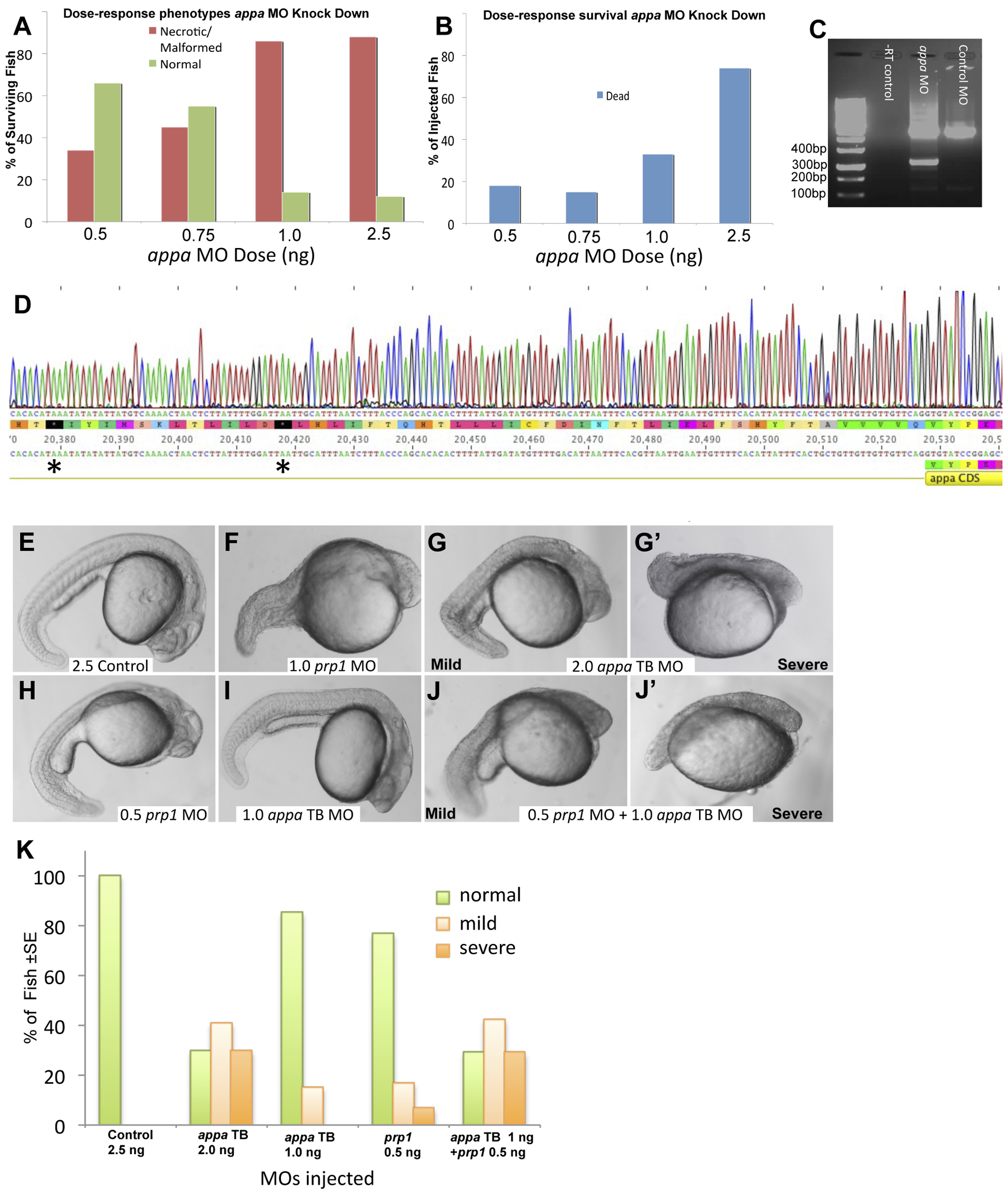

Supplement: Figure S1 — appa morpholino (MO) injection leads to a dose-dependent disruption in appa mRNA processing. A. Zebrafish embryos were injected with increasing doses of appa MO and scored for malformations and CNS cell death. B. Same experiments as panel A revealed doses that were toxic to the developing fish. C. appa splice block MO is efficacious, as it leads to disruption of appa mRNA. RNA was isolated from fish injected with appa MO, an equivalent dose of control MO, and subjected to RT-PCR. Fish injected with appa MO show a band at ∼300 bp corresponding to mRNA with intron 2 retained. This band is absent in when fish are injected with the control MO, or when standard Taq is used in place of reverse-transcriptase. D. Sequencing of the aforementioned ∼300 bp band confirms the retention of intron 2–3 in mature mRNA, and confirms our MO produces a truncated protein. Our sequence (top) was an exact match to zebrafish genomic clone NW_003336735 (bottom). Translation of the sequence, immediately 5′ of appa exon 3 (annotated in yellow at bottom right), predicts two termination codons (black, *). E–J. A second MO reagent against appa was used to test specificity of the phenotypes observed. Designed against a disparate portion of the gene, the 5′UTR and thus is a translation blocking (TB) MO. The efficacy of this MO is demonstrated in Fig. 1M and 1N. It produced mild and severe phenotypes (G and G′) indistinguishable from the splice blocking appa MO we primarily use in this work. The low dose of appa TB MO also showed a genetic interaction with the low dose of prp1 MO, equivalent to results in Fig. 3 (J, J′). MO dose is indicated on panels. K. Quantification of appa-TB MO demonstrates a dose-dependant effect by itself and an additive effect with prp1 MO during concerted delivery at sub-effective doses. Colour coding in the histogram is as per Fig. 2. (TIF) [file pone.0051305.s001.tif]

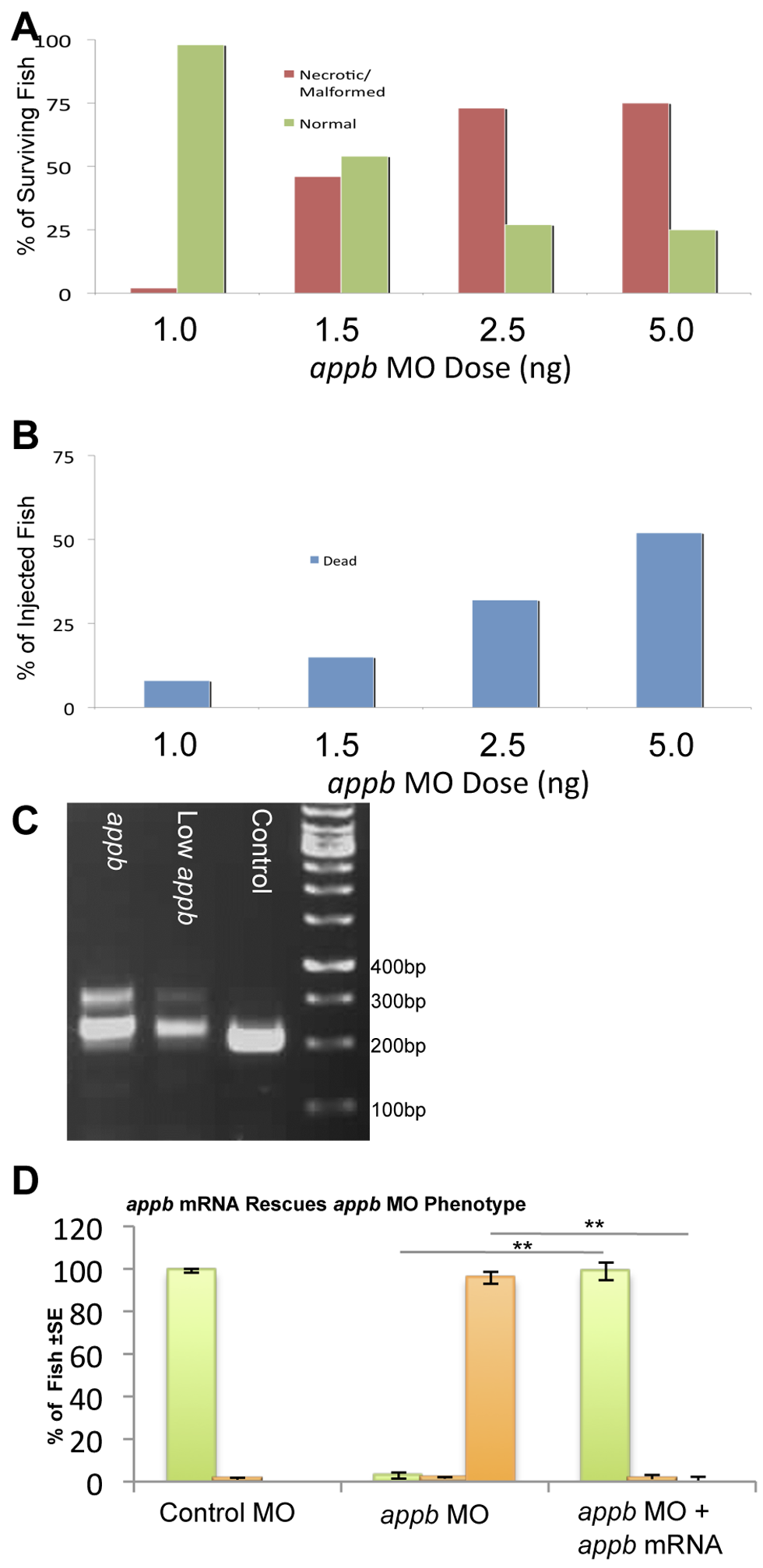

Supplement: Figure S2 — appb morpholino (MO) injection leads to a dose-dependent disruption in appb mRNA processing. A. Zebrafish embryos were injected with increasing doses of appb MO and screened based on presence of morphological malformations and CNS cell death. B. The same experiments as in panel A revealed doses that were toxic to the developing fish. C. appb splice block MO is efficacious, as it leads to disruption of appb mRNA. RNA was isolated from fish injected with 2.5 ng appb MO, 1.0 ng appb MO, or an equivalent dose of control MO, and subjected to RT-PCR. Fish injected with 2.5 ng of the appb MO show a band at ∼300 bp corresponding to retention of intron 3–4 in mRNA. This band is reduced when the dose of the MO is reduced, and absent when fish are injected with the control MO. Sequencing of the band confirmed the retention of intron 3–4 in mature mRNA, and predicted STOP codons in the modified mRNA. D. Embryos injected with the appb MO alone or with 200 pg of cognate appb mRNA. The instance of fish displaying a severe phenotype was significantly reduced and the number of normal fish was significantly increased upon inclusion of appb mRNA. **p<0.01. Colour coding in the histogram D is as per Fig. 2 and Fig. S1. (TIF) [file pone.0051305.s002.tif]

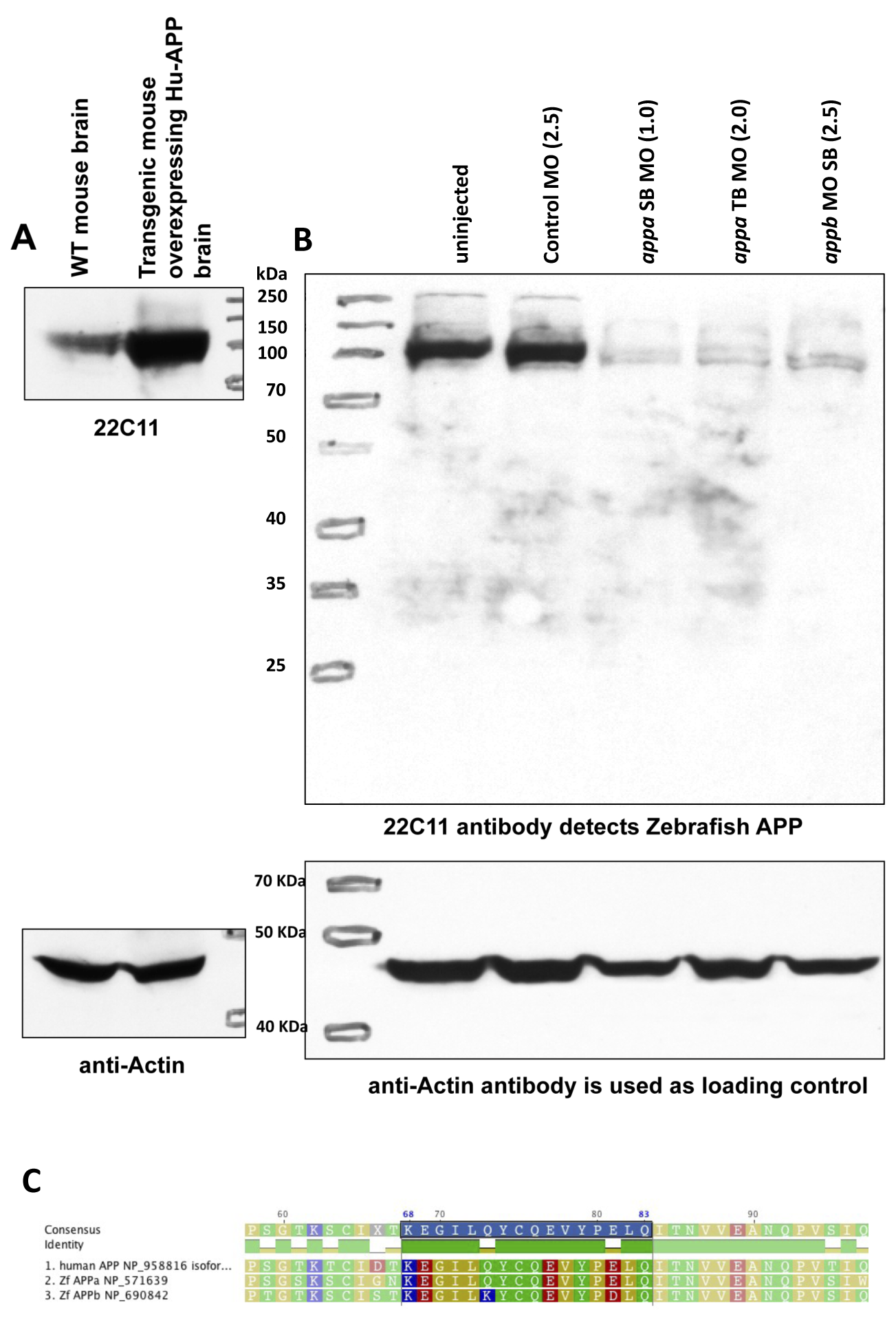

Supplement: Figure S3 — Efficacy of appa and appb MO’s assessed by western blot. MO knockdown reagents were assessed by Western blot to quantify protein abundance. Parts of this data appear in Fig. 1M. A. The size of the APP-immunoreactive bands in wild type mouse brain or from TgCRND8 mouse brains overexpressing human APP as detected with the antibody 22C11. B. Zebrafish App proteins are detected with 22C11 and the bands are indistinguishable from mammalian APP observed in panel A. Knockdown of zebrafish appa or appb gene products with various MO reagents (See Fig. 1, doses in nanograms are presented in brackets at the top of the figure) results in a significant reduction of APP immunoreactivity as normalized to β-actin levels and compared to control MO-injected fish. Smaller protein products that might be predicted to have a dominant effect following injection of splice blocking MOs are not detectable (predicted size of MO-altered proteins are 10 and 20 kDA for Appa and Appb, respectively). C. The 22C11 epitope (highlighted in blue) is conserved between human (top line) and zebrafish (Zf) proteins Appa (16/16 residues identical) and Appb (14/16 residues identical, 15/16 residues with conserved identity). Identity of the region is represented on the graph above the alignment with green showing perfect identity and amber showing mismatches. (TIF) [file pone.0051305.s003.tif]

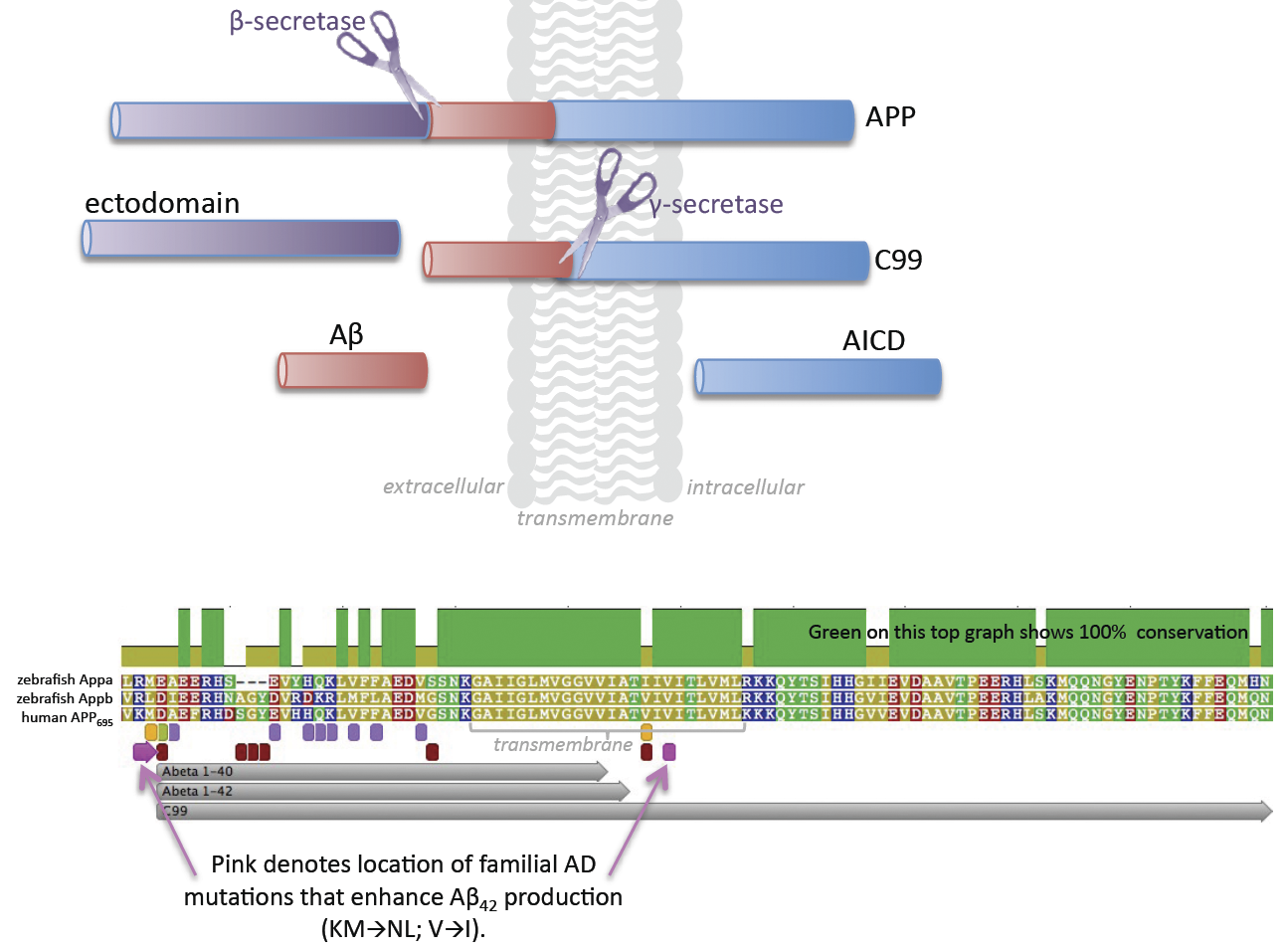

Supplement: Figure S4 — Conservation of App between zebrafish paralogs and human APP. Amyloid β Precursor Protein (APP) is processed to Aβ (red), the major protein constituent of plaques in AD, by sequential enzyme cleavage. Zebrafish have two gene paralogues, appa & appb, wherein most residues of C99 at least one of them is a perfect match to human. Human APP is able to replace Appa in this interaction, Appa is more similar to human (purple boxes, red boxes show where Appb is more similar). Residues responsible for familial AD mutations (pink) are conserved in all three proteins. (TIF) [file pone.0051305.s004.tif]

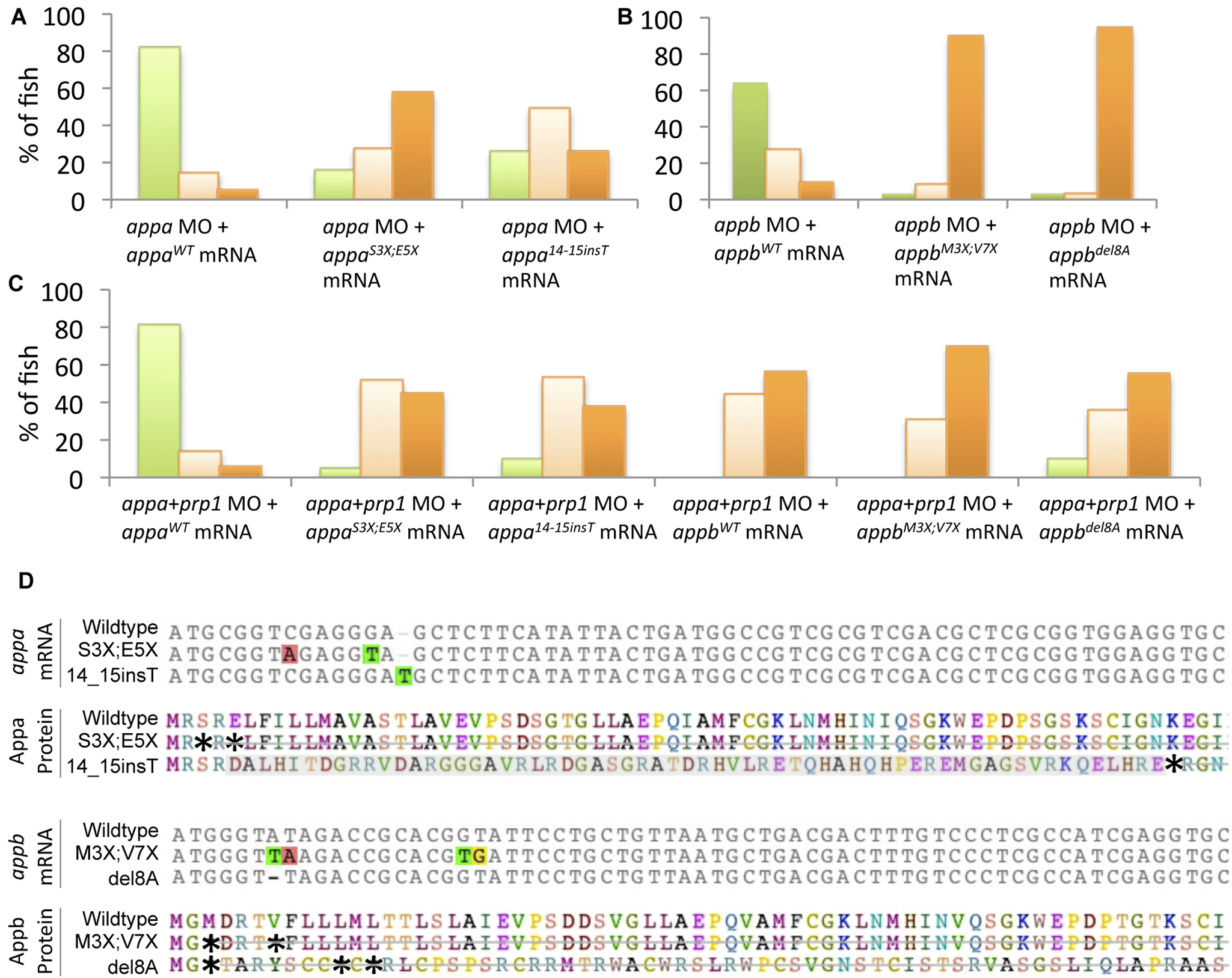

Supplement: Figure S5 — appa and appb mRNAs that rescue observed phenotypes must be translated to have their effect. The phenotypes produced by knockdown of appa or appb can be rescued by co-injection of wild type appa or appb mRNA, respectively (Fig. 1–2, S1–S2, and left-hand data set of panels A & B here), but not by mutant mRNAs. A, B. Assessment of mutant mRNAs. Mutant mRNAs possessing point mutations in the start of the coding region fail to rescue phenotypes that are rescued by wildtype (WT) mRNA. C. The same result is found regarding the ability (or lack thereof) of these mRNAs to rescue the joint knockdown of prp1 and appa (part of Fig. 2L is replicated here for clarity). D. Two separate alterations to each of appa and appb were made, with a goal of making subtle alterations to the mRNA molecule (<5 basepairs changed in the ∼2200 bp molecule) that ablate capacity to encode a functional full-length protein. In appa, we changed two basepairs (highlighted) at the start of the CDS that are predicted to create large changes in the protein by creating stop codons (S3X;E5X) (asterisks in protein represent stop codons) inducing a truncation. Alternatively, we modified the appa CDS by a single basepair insertion (14_15 insT) inducing a frameshift of 72 residues (grey shading) before a premature stop codon, and with only one of 72 residues having sequence identity with the parental wt mRNA. Similarly in appb, changing four base pairs created stop codons (M3X;V7X) and a truncated protein. We altered the appb mRNA in a second way, inducing a single basepair deletion (del8A) to create stop codons and a truncated protein. Stop codons are represented by asterisks, and residues N-terminal to stop codons are emphasized with strikethrough. Colour coding of histograms as per Fig. 2. (TIF) [file pone.0051305.s005.tif]

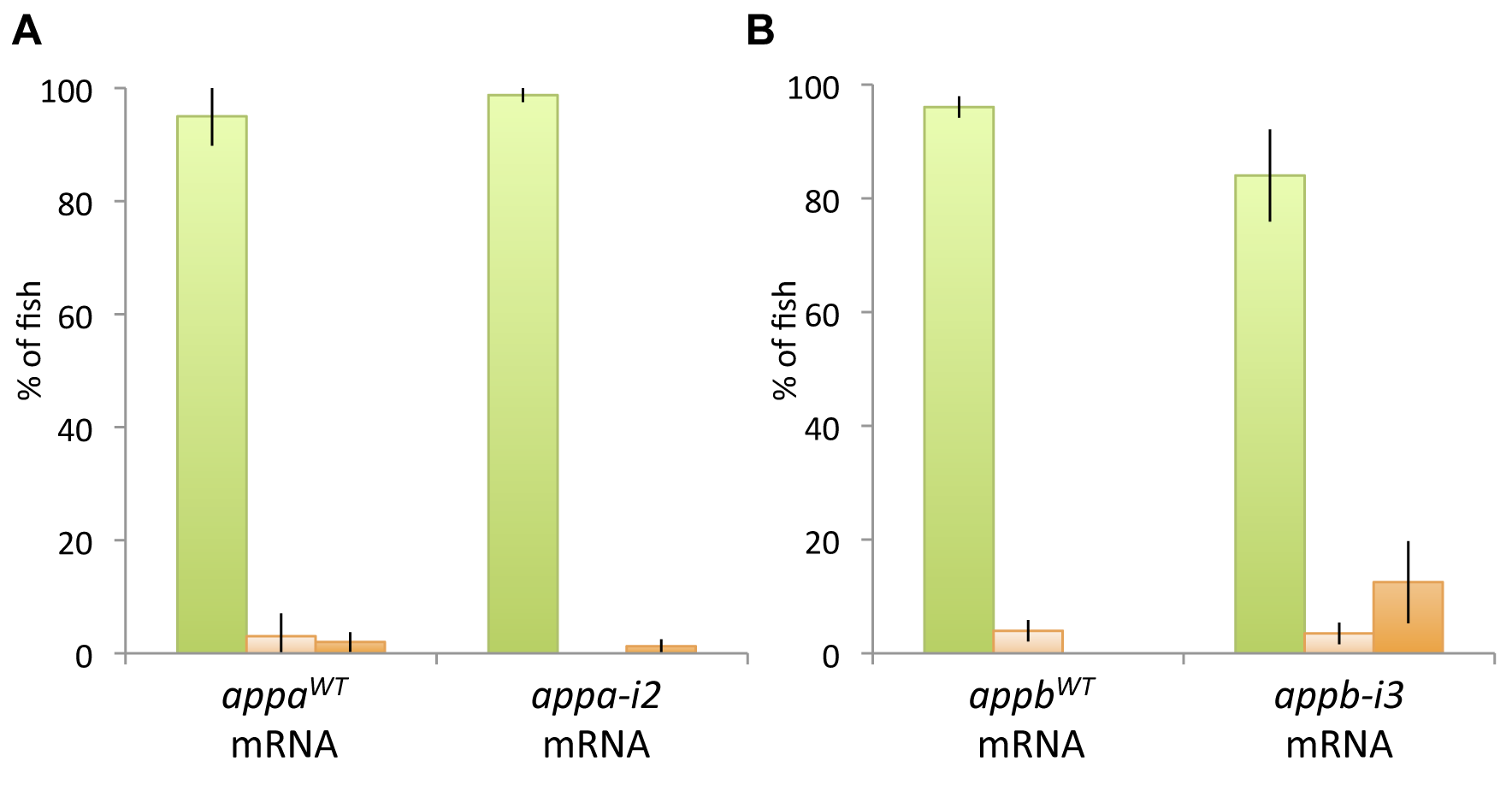

Supplement: Figure S6 — mRNAs envisaged following injection of splice blocking morpholinos do not have a dominant effect. Injection of the splice blocking MOs leads to inappropriate retention of the adjacent intron (with STOP codons); this is predicted to encode a truncated protein along with a portion of the retained intron. This altered mRNA may be degraded, but the kinetics are unknown so potential dominant effects of the predicted protein were tested, by delivering an mRNA encoding the truncated mRNA with retained intron. A. Delivery of mRNA encoding the first two exons of appa plus intron 2 (appa-i2, N = 3 trials, n = 114 fish). B. Delivery of mRNA encoding the first three exons of appb plus intron 3 (appb-i3, N = 2 trials, n = 91 fish). Data obtained from injecting mRNAs encoding the full length appa and appb proteins are presented for ease of comparison, replicated from Figure 2E and F, respectively. No dominant effect or significant change in phenotypes was observed following injection of appa-i2 or appb-i3 mRNA compared to injecting the cognate full length mRNA, or compared to injecting control MOs, or compared to uninjected fish. Thus MO injection likely leads to the phenotypes observed through reduction of protein abundance, and is not due to dominant effects of products from mis-spliced mRNAs. Colour coding of histograms as per Fig. 2. (TIF) [file pone.0051305.s006.tif]

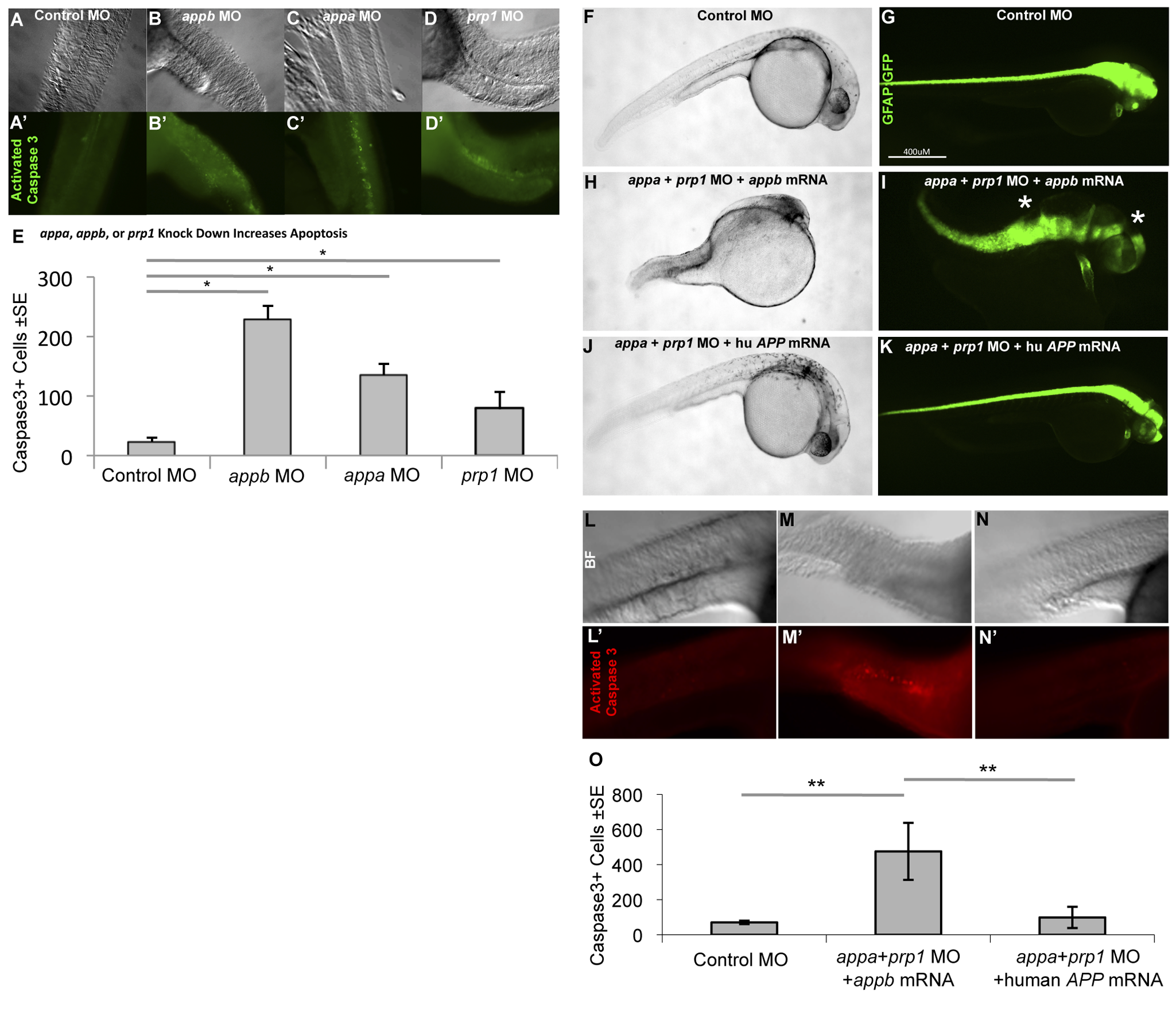

Supplement: Figure S7 — High doses of MOs affect apoptotic cell death, and Human APP rescues apoptotic cell death. High doses of prp1, appa or appb MO are used here to show their individual effects on apoptotic cell death. Some combinations of these MOs co-injected at low doses show that these MOs can synergize to produce this effect (Fig. 4). Apoptosis levels are increased when appa, appb, or prp1 mRNA is disrupted (A–D). Brightfield images of the area above the yolk sac extension of fish injected with effective doses of control, appb, appa and prp1 (A–D, respectively) MOs. Compared with control fish, prominent anti-activated caspase 3 staining is apparent in appb, appa, or prp1 MO injected fish (A′–D′, respectively). E. Number of caspase 3 positive cells were counted above the yolk sac extension in fish treated as per those in A–D. N = 5. Panels F–K show examples of human APP rescuing the concerted appa plus prp1 knockdown. appb is not able to rescue the phenotype in wildtype fish, nor in transgenic fish labelling the CNS with GFP (H & I, respectively, compare to F & G) as we noted in Fig. 3K and here serves as a negative control. A noticeable lack of GFP was apparent along portions of the CNS (* in panel I). Human APP is able to rescue these phenotypes (J, K). Panels L–N show the yolk-sac extension of fish in F, H & J. L′–N′ show examples of activated caspase labelling during rescue with human APP. The latter treatments were quantified in O. N = 5. * = P<0.05. ** = P<0.01. (TIF) [file pone.0051305.s007.tif]
