# Supplementary material for: Amyloid Beta Precursor Protein and Prion Protein Have a Conserved Interaction Affecting Cell Adhesion and CNS Development
Source: PLoS One. 2012 Dec 7;7(12):e51305. doi: 10.1371/journal.pone.0051305 (PMC3517466; doi:10.1371/journal.pone.0051305)
Supplement: Protocol S1 — Supplemental description of Methods. (DOCX) [file pone.0051305.s008.docx]

**Protocol S1**

**Supplemental Methods:**

**mRNA rescue experiments**: *appa*, *appb*, *prp1, prp2* and *sho1* cDNAs (Accession numbers JQ994487-JQ994491 associated with ZFIN numbers ZDB-GENE-000616-13, ZDB-GENE-020220-1, ZDB-GENE-041221-2, ZDB-GENE-041221-3, ZDB-GENE-031110-1) were cloned from wild type zebrafish into a PCS2+ or pCR2.1TOPO vector (primers in Table 1), and sequenced to confirm identity. These cDNAs were linearized with appropriate restriction endonucleases and used to produce mRNA using the mMessage SP6 kit (Ambion Cat# AM1340). Human *APP_695_* cDNAs were cloned into PCS2+ vector, linearized with *Not*1 and transcribed for 3 hours using the mMessage SP6 kit (Ambion Cat# AM1340). Site-directed mutagenesis was performed using Quikchange Site-directed Mutagenesis Kit (Stratagene) using primers in Table 1. To test for dominant effects, mRNAs matching the predicted variants following splice block MO injection were ordered as custom minigenes (Integrated DNA Technologies, San Diego, CA) and cloned into pCS2+ using engineered restriction sites. For *appa*, this included exons 1 and 2, along with intron 2-3 (= 279 bp CDS, denoted ‘*appa-i2*’); for *appb*, this included exons 1-3, along with intron 3-4 (= 538 bp CDS, denoted ‘*appb-i3*’). mRNA for *appa-i2* and *appb-i3* were injected at doses similar to but exceeding those of the cognate full-length mRNA (1.3-fold and 2.2-fold more, respectively) to best assure detection of dominant effects, if any. In both cases the intronic sequence cloned was the 5’ portion up to and including the first endogenous in-frame stop codon.

mRNA prepared for injections was quantified using a GE NanoVue spectrophotometer and/or using a BioAnalyzer, electrophoresed on a 1% nuclease free agarose gel to check their integrity, and stored at -80°C until use. mRNA was then dissolved in injection solution without morpholino and optimal dosage determined using a dose response curve. For rescue experiments mRNA was dissolved in the appropriate morpholino containing injection solution and injected along with the morpholino. Zebrafish were staged to 24 hpf and screened based on body morphology and appearance of CNS cell death. Observer was blinded to treatment groups during screening of all phenotypes.

**Determining MO Reagent Specificity by Quantifying Phenotypes during mRNA Rescue:** During an initial screen we quantified fish showing morphological deficits along with visually apparent cell death at 24 hours post-fertilization (hpf), classified as “necrotic/malformed” (Fig. S1A, S2A). At low *appa* MO doses of 0.5ng and 0.75ng a small percentage (~20-40%, Table 1, Fig. S1A) of fish displayed a mild phenotype consisting of slight body and CNS malformations, whereas at a dose of 1.0ng 86% (Fig. S1A) of living fish displayed a phenotype of body and CNS malformations, with the majority of those also displaying overt CNS cell death (Fig. 1C, S1A). Fish injected with increasing doses of MO against *appb* showed an increased occurrence of a neurodevelopmental phenotype that closely mimicked the phenotype apparent from *APPa* knockdown (Fig. 1C,G). Fish injected with low doses of the *appb* MO (1ng) showed no apparent phenotype, while at the “effective dose” of 2.5 ng the percentage of fish displaying a malformed CNS with cell death rose to 73% (Table 1) (Fig. S2). For the remainder of the *app* MO injection experiments fish that were morphologically disrupted but had no detectable cell death were classified as “mild” and fish that were both morphologically disrupted and had obvious cell death were classified as “severe” (Fig. 1). To further confirm specificity of the *appa* and *appb* MOs, fish were co-injected with 200 pg of cognate zebrafish mRNA. Injection of *appa* mRNA alone did not cause any significant phenotype (Fig. S1C, Table1) while co-injection of *appa* mRNA along with the *appa* MO significantly increased the percentage of “normal” fish compared to those that only received the *APPa* MO (79±12% vs 24±24%, P<0.05, Figs. 1 and 2F, Table 1). The percentage of fish that displayed a “severe” phenotype was also significantly reduced to 8±12% (P<0.05) in fish receiving both the *appa* MO and mRNA opposed to 45±45% of those that received the *appa* MO only. Co-injection of *appb* mRNA significantly increased the percentage of “normal” fish compared to those that only received the *APPb* MO (99±4% vs 3±1%, P<0.01) (Fig. S2, Table 1). The percentage of fish that displayed a “severe” phenotype was also significantly reduced in fish receiving both the *appb* MO and mRNA opposed to those that received the *appb* MO only (0±2% vs 96±3%, Fig. S2 and Table 1).

**Determining MO Reagent Efficacy by Western Blot Analysis:** We quantified App protein abundance following injection of *app* MOs using Western blots. Similar analyses have previously demonstrated efficacy of the *prp1* MO we used [27]. MOs were injected at 2.5, 1, 2, and 2.5 ng/embryo for control, *appa*_SB, *appa*_TB and *appb* MO reagents as described above. Three biological replicates were performed by injecting all four MOs on three separate days. In one trial, the phenol red in the injection solution was replaced with 2.5 mg/mL dextran conjugated to AlexaFluor488 (Invitrogen D22910), and we selected embryos for processing that displayed green fluorescence (~90-98% of injected fish), indicating a successful delivery of the injection solution. This refinement improved our ability to detect reductions in protein abundance for some MOs, but did not change the overall trend nor statistical significance of our results. Both injected and un-injected embryos (n≅60 per group) were manually dechorionated at 3 dpf and disrupted by repeated pipetting in a buffer (55 mM NaCl, 1.8 mM KCl and 1.25 mM NaHCO_3_) to remove the yolk. Cells were pelleted and subsequently lysed in lysis buffer (20 mM HEPES pH7.6, 0.5 M NaCl, 1.5 mM MgCl_2_, 1 mM DTT, 0.1% Triton X-100, 20% glycerol). Lysates were separated on a 10% SDS-PAGE gel, blocked with 5**%** milk in Tris-buffer saline with tween-20 (TBST) and immunoblotted with anti-APP antibody 22C11 (Millipore MAB348SP) diluted 1:5000 in 5**%** milk in TBST. The anti-APP antibody 22C11 is a good candidate to detect both Appa & Appb in zebrafish (Fig. S3) because 22C11 binds amino acids 66-81 of human APP [83] and this epitope, comprised of 16 amino acids, is 100% identical in zebrafish Appa, whereas in Appb there is one conservative (E🡪D) and one non-conservative (Q🡪K) amino acid difference (Fig. S3). Immunoreactivity was detected with 1:5000 goat-anti-mouse-HRP (Jackson Immunoresearch 115-035-003) and developed with enhanced chemiluminiscent reagent (Pierce). Blots were reprobed with 1:5000 anti-β-actin (Sigma A2066; ZFIN ID: ZDB-ATB-100427-2) and 1:5000 goat-anti-rabbit-HRP (Jackson Immunoresearch). Densitometry of bands was performed using ImageJ (National Institutes of Health, Bethesda MD). Ratio of APP to β-actin immunoreactivity was calculated, and means and standard error of the three biological replicates were plotted using Excel (Microsoft, WA).

**Reverse Transcriptase-Polymerase Chain Reaction**: RNA was purified from 24 hpf embryos stored in RNAlater (Qiagen Cat# 76104) using the RNeasy kit (Qiagen Cat# 74104). *appa* morpholino efficacy was determined using primers designed to bind intron 2 and exon 8 of zebrafish *appa* (Table 2). *appb* morpholino efficacy was determined using primers designed to bind exon 3 and exon 6 of zebrafish *appb* (Table 2). For all RT reactions PCR was also run on extracted RNA with standard Taq polymerase in place of reverse transcriptase to confirm absence of genomic DNA contamination.

Following determination of an effective MO dose, we confirmed by RT-PCR that the *appa* MO led to retention of intron 2-3 and sequencing revealed this intron contained 5 in-frame stop codons (Fig. S1). Likewise, RT-PCR of *appb* MO injected fish showed retention of intron 3-4 in mature mRNA and sequencing of this mRNA revealed the presence of 3 in frame stop codons (Fig. S2).

**Immunohistochemistry**: 24 hpf zebrafish were fixed in 4% paraformaldehyde in PBS (phosphate buffered saline, pH 7.4) with 5% sucrose overnight and rinsed for 20 min in PBS with 5% sucrose (3 times), washed 5 min in water with 1% Tween, washed 7 min in -20^o^C acetone, blocked for 1hour at RT in PBS with 10% normal goat serum, incubated in 1:500 anti-activated caspase 3 antibody (BD Biosciences Cat #559565; previously validated in zebrafish- ZFIN ID: ZDB-ATB-081107-1) in PBS with 1% Tween, 1% DMSO, 1% Triton and 2% NGS overnight at 4^o^C. Samples were then washed for 20 min in PBS containing 1% Tween, 1% DMSO, and 1% Triton twice then incubated overnight in secondary antibody (1:1000 anti-Rabbit-AlexaFluor-488 or -555, or anti-Mouse-AlexaFluor-488, Invitrogen cat#s A21441, A31572, A21202, A31570) in PBS with 1% Tween 1% DMSO 1% Triton and 2% NGS, and washed for 20 min in PBS containing 1% Tween, 1% DMSO, and 1% Triton (3 times). After the final wash zebrafish were placed in anti-fade mounting media (Invitrogen cat# S36937) and visualized on a Nikon 90i microscope. Activated-caspase 3-positive cells were imaged and quantified above the yolk sac extension of the fish as per past methods [36].

**Immunoprecipitation:** To determine if human APP and PrP physically interact in protein complexes, we expressed full-length human PrP and/or human APP_695_ in mouse N2a cells and used antibodies against human PrP and APP that do not detect mouse homologs. This combination of mouse cells (including our generation of cells expressing human APP, human PrP, both or neither) and human-specific antibodies allowed us to unequivocally assess reagent specificity by having negative control cells that lacked APP and/or PrP immunoreactivity. Two groups of N2a cells were used, those that are stably transfected with *APP_695_* [84] or an unmodified N2a cell clone. The former were a gift of Dr. Gopal Thinakaran, (University of Chicago, USA). Lipofectamine (Invitrogen) was used to transfect both groups of cells with either empty vector pcDNA3.1 or with human *PRNP****^M129^*** cloned into this same vector using *Bam*HI and *Xba*I sites. Transfections were completed in 10 cm dishes with 24 μg of DNA per transfection (as suggested by manufacturer). Two days after transfection the cells were harvested and processed for immunoprecipitation using methods similar to those we have published previously [85]. The cells were pelleted by centrifugation at 300X*g* for 10 minutes and resuspended in 300 µl of lysis buffer (20 mM HEPES [pH 7.6], 0.5 M NaCl, 1.5 mM MgCl2, 1mM DTT, 0.1% Triton X-100, and 20% glycerol). Protein from these lysates was quantified by colourimetry using BioRad Protein Assay Dye Reagent (catalogue # 500-0006) and added to radioimmunoprecipitation assay buffer (RIPA: 1× PBS, 1% (v/v) IGEPAL CA-630, 0.5% (w/v) sodium deoxycholate, 0.1% (w/v) SDS) to a final concentration of 300 μg protein / mL RIPA with protein A+G-agarose beads (25 μL bed volume, Santa Cruz Biotechnology Inc., CA, USA, # sc-2003). The beads had been pretreated with bovine serum albumin (BSA) by washing 3X in PBS and then incubating with 1% BSA in PBS for 1 hour at 4°C; this was followed by centrifugation at 1000X*g* and maintenance at 4°C in an equal volume of RIPA buffer. Protein lysates were precleared by incubation with pretreated beads for an hour at 4°C, and subsequently incubated overnight with 1 µg of polyclonal anti-APP antibody raised in rabbit (LifeSpan Biosystems, LS-B691) at 4 °C with continuous rotation. These anti-APP-antibody-treated cell lysates were incubated with a 25 µL bed volume of pretreated beads for 1.5 hours at 4°C with continuous rotation. Following four washes with RIPA buffer the protein complexes were eluted from the beads in SDS-PAGE loading buffer. These complexes and input fractions were separated by 10% SDS-PAGE and subjected to immunoblot analysis with a mouse monoclonal antibody against prion protein (3F4 at 1:10000, Covance, Princeton NJ, SIG-39600) that binds an epitope in human PrP that is absent from mouse PrP. Antibodies were diluted in 5% milk in TBST. Immunoreactivity was detected with 1:5000 goat-anti-mouse-HRP (Jackson Immunoresearch) and developed with enhanced chemiluminiscent reagent (Pierce). The blots of inputs were subsequently probed with anti-APP antibody 6E10 (1:3000, Covance, SIG-39347). Input fraction represented 7% of the protein extract used for all immunoprecipitation experiments.

**Aggregation Assays**: One-cell stage zebrafish embryos were injected with morpholino solutions as above but containing 2.5 μL of Dextran conjugated to AlexaFluor-488 or -555 (Invitrogen D1817, D22910) in place of phenol red. Embryos were incubated at 28.5^o^C until dome stage was reached. Embryos were then manually dechorionated and placed on agarose plates containing E3 media with 1:200 Penicillin/Streptomycin antibiotic. Yolk was manually removed from the cells using insect pins and yolk free cells were placed in a 1.5 mL microcentrifuge tube containing 1 mL Ca^2+^ free Ringers solution (116 mM NaCl, 2.9 mM KCl, 5.0 mM HEPES, pH 7.2). Cells were dissociated by pipetting repeatedly with a 200 μL pipette for 30 seconds. A portion of these cells were assessed at this point to check that dissociation had occurred. Cells were centrifuged at 550xg for 30 seconds and supernatant removed. 600 μL high calcium Ringers solution (116 mM NaCl, 2.9 mM KCl, 10 mM CaCl2, 5 mM HEPES, pH 7.2) was then added to cells and cells were incubated at 28.5^o^C for 1hr in a standard 1.5mL microcentrifuge tube. 200 μl of solution was aliquoted in triplicate into wells of 96 well plate and imaged on a GE IN cell 2000 analyzer. Exposure was manually adjusted and 9 images (fields) per well were collected at 20X magnification. Analysis of images was done using the GE investigator software package; exposure and contrast were equalized across wells, and the mean size of a single cell calculated. Any contiguous fluorescent signal 10X larger in area than a single cell (see results) was then defined as an aggregate. Multi-level object linking was used to determine the total number of cells within these aggregates along with the total number of cells in the well. Data was then manually processed in Microsoft Excel; to avoid ambiguous determination of aggregates only aggregates containing more than 10 cells in direct physical contact were counted and this number in proportion to the total number of cells in the well was used to calculate the percentage of cells in aggregates.

**Image manipulation:** All images were taken on Nikon 90*i* microscope using QImaging RETIGA 2000R camera. Images were rotated, cropped and brightness adjusted in Photoshop CS5 and further cropped/assembled in Powerpoint 2008. No other alterations were made.

**Statistics:** All statistics except Western blot results were performed in SPSS 17.0 using one-way ANOVA with Bonferroni correction and Tukey post hoc tests, or Mann-Whitney U test. Western blot results were analyzed by ANOVA using Systat12 with Tukey post-hoc pairwise tests. Significance was set to 0.05 (*) or 0.01 (**).
